# Sulfate-reducing bacteria in acid mine drainage: ecological constraints, microbial networks, and functional persistence

**DOI:** 10.3389/fmicb.2026.1910720

**Published:** 2026-08-11

**Authors:** Rui Xiao, Jiaoyang Liu, Cunzeng Li, Yang Peng, Penghua Hu, Guoping Jiang

**Affiliations:** 1 Beijing Research Institute of Chemical Engineering and Metallurgy, China National Nuclear Corporation, Beijing, China; 2 College for Elite Engineers, China University of Geosciences, Wuhan, China; 3 School of Minerals Processing and Bioengineering, Central South University, Changsha, China

**Keywords:** acid mine drainage (AMD), bioremediation, functional persistence, iron–sulfur cycling, microbial networks, sulfate-reducing bacteria (SRB)

## Abstract

Sulfate-reducing bacteria (SRB) are key functional microorganisms in the bioremediation of acid mine drainage (AMD), simultaneously removing sulfate, generating alkalinity, and precipitating metal sulfides via dissimilatory sulfate reduction. AMD is typically characterized by low pH, high sulfate and metal concentrations, and limited organic carbon, imposing persistent stress on SRB growth, metabolism, and function. Environmental stressors include acidic conditions, heavy metal toxicity, low-temperature stress, and electron-donor limitation. These stressors impair membrane stability, enzymatic activity, and cellular energy conservation. Their combined effects increase maintenance requirements while limiting energy acquisition, ultimately reducing the range of environmental conditions under which SRB can sustain sulfate reduction. At the community level, SRB function is further modulated by complex microbial networks. Various functional groups, including fermenters, methanogens, and sulfur- and iron-cycling microorganisms, interact with SRB through cooperative, competitive, and regulatory processes that influence electron transfer, carbon turnover, and iron–sulfur transformations. Sulfate reduction in AMD depends on these community-level interactions, which can be disrupted under environmental stress and may reduce the stability of sulfate-reducing communities. Engineering strategies such as slow-release carbon supplementation, pH microenvironment optimization, conductive material amendment, mineral–microbe interface regulation, and immobilized reactor design can enhance SRB persistence by stabilizing the extracellular microenvironment, regulating electron flow, and spatially decoupling metabolic and mineralization interfaces. This review highlights how environmental stressors and microbial networks jointly regulate SRB function, emphasizes the roles of metabolic niche constraints and community resilience, and provides mechanistic insights for improving the stability and practical performance of SRB-based AMD treatment systems.

## 1 Introduction

Acid mine drainage (AMD) represents a persistent and complex environmental challenge associated with mining activities, arising from the oxidative dissolution of sulfide minerals under the combined influence of oxygen, water, and microorganisms. AMD is typically characterized by low pH, high sulfate concentrations, and elevated levels of toxic metals and metalloids, leading to water acidification, soil functional degradation, and contaminant accumulation through food webs, thereby posing long-term ecological risks in mining-impacted regions (Skousen et al., 2017; Jennings et al., 2025; Pérez-López et al., 2025). Effective remediation therefore requires the simultaneous neutralization of acidity, reduction of sulfate, and stabilization of metal contaminants.

Conventional AMD treatments primarily rely on physicochemical approaches such as lime neutralization and chemical precipitation. While widely implemented, these methods often incur high reagent and operational costs, generate large volumes of secondary sludge, and achieve limited sulfate removal, restricting their sustainability in long-term applications (Mosai et al., 2024; Chitransh and Mondal, 2025). In contrast, bioremediation strategies based on sulfate-reducing bacteria (SRB) offer an environmentally friendly alternative. Through dissimilatory sulfate reduction, SRB simultaneously generate alkalinity and produce sulfide that precipitates metals, enabling coordinated mitigation of multiple AMD stressors (Rambabu et al., 2020; Laroche et al., 2023). The overall sulfate reduction process can be represented as:


$${\text{SO}}_{4}^{2-}+8{\text{e}}^{-}+10{\text{H}}^{+}\to {\text{H}}_{2}\text{S}+4{\text{H}}_{2}\text{O}$$

This reaction highlights the coupling between electron transfer, proton consumption, and sulfide production in AMD systems.

However, the extreme geochemical conditions of AMD exert strong selective pressures on microbial communities, often reducing biodiversity while promoting highly specialized functional networks dominated by tightly coupled iron, sulfur, and carbon biogeochemical cycles (Méndez-García et al., 2015; Du et al., 2022; Budania and Dangayach, 2023). Within these ecosystems, SRB are central to sulfur cycling, metal transformation, and local redox modulation. Their functional persistence depends not only on intrinsic physiological tolerance but also on community-level interactions, including electron flow, carbon cross-feeding, and microenvironmental redox regulation (Zhang et al., 2022; Mafane et al., 2025).

Sustaining SRB activity under AMD conditions remains challenging because multiple stressors occur simultaneously. Acidic stress, metal toxicity, low-temperature constraints, and electron-donor limitation increase maintenance energy requirements and restrict energy acquisition, thereby reducing the conditions under which SRB can sustain sulfate reduction (Xu and Chen, 2020; Cun et al., 2024; Valdez-Nuñez et al., 2024; Karnachuk et al., 2025). Most studies have focused on responses to individual stressors, whereas the mechanistic basis of SRB functional stability under combined environmental constraints remains poorly resolved (Cun et al., 2024; Zhang et al., 2026). Furthermore, the roles of supporting microbial guilds, including fermentative microorganisms, iron-reducing bacteria, and sulfur-disproportionating bacteria, have largely been investigated separately from those of extracellular engineering factors such as conductive materials, mineral interfaces, and immobilization carriers. How these biological and abiotic components interact to regulate SRB metabolism and interspecies electron transfer in AMD systems remains insufficiently understood (Dong et al., 2020; Gou et al., 2024; Licona et al., 2025; Ren et al., 2025; Shu and Wu, 2025; Zhu et al., 2026).

Because SRB performance under AMD depends on the balance between energy acquisition and cellular maintenance costs, we use the term metabolic niche as a conceptual framework to describe the energetic and physiological conditions required for sustained sulfate reduction. Accordingly, metabolic niche contraction refers here to the progressive reduction in the range of energetic and physiological conditions that support sustained sulfate reduction under multiple environmental stressors. This review synthesizes current understanding of the environmental constraints and microbial interactions that regulate SRB function in AMD remediation. We further examine strategies to sustain long-term SRB activity, including electron donor supplementation, pH buffering, enhancement of extracellular electron transfer, mineral–microbe interactions, and immobilization approaches. Integrating these perspectives provides mechanistic insight into SRB stability under extreme conditions and informs the design of resilient, sustainable AMD bioremediation systems.

## 2 Environmental constraints on SRB metabolic niches

The extreme nature of AMD arises from the combined effects of multiple physicochemical stressors. Low pH, low temperature, elevated metal concentrations, and organic carbon limitation suppress SRB activity through distinct but interconnected mechanisms that collectively constrain sulfate reduction. Table 1 summarizes the major environmental constraints, their inhibitory mechanisms, representative adaptive responses, and the current status of supporting evidence. Throughout the following discussion, we distinguish mechanisms directly demonstrated in SRB from observations obtained in particular SRB strains or sulfate-reducing consortia and from hypotheses inferred from other microorganisms that remain to be validated in AMD-associated SRB. Representative acidophilic and acid-tolerant SRB reported from AMD and related mining environments are summarized in Table 2. Unless otherwise specified, the physiological classifications used throughout this section are based on experimentally reported growth-pH ranges rather than environmental occurrence alone.

**Table 1:** Environmental constraints shaping the ecological niche of SRB in AMD systems and current evidence status of adaptive mechanisms.

Stress factor	Major inhibitory mechanism	Adaptive response/mechanism	Evidence status	References
Low pH	Proton influx and intracellular acidification; increased energetic cost of pH homeostasis	K^+^ transport regulation (*kdpABC*), membrane lipid remodeling, ion homeostasis, and proton extrusion	Directly demonstrated in specific acidophilic SRB/aSRB strains; broader ion-homeostasis mechanisms require further validation	Sánchez-Andrea et al., 2022; Egas et al., 2024, 2025; Zhang et al., 2026
Low temperature	Reduced membrane fluidity and enzyme activity	Membrane lipid adjustment, cold-shock response, and potential roles of compatible solutes and EPS-mediated protection	Cold adaptation is demonstrated in psychrophilic/psychrotolerant SRB, whereas several molecular mechanisms remain inferred from other microorganisms and require validation in SRB	Wu et al., 2023; Mafane et al., 2025; Xiao et al., 2025
Heavy metals	Enzyme inhibition, oxidative stress, and membrane damage	Sulfide precipitation, EPS-associated metal binding, and metal-responsive transport systems	Sulfide precipitation is strongly supported; EPS-mediated protection and metal transport are supported in specific SRB systems, whereas RND-mediated resistance and Fe–S enzyme protection remain insufficiently validated	Qian et al., 2018; Capeness et al., 2019; Saxena et al., 2025a,b
Organic carbon scarcity and electron-donor limitation	Electron donor deficiency and reduced energy availability	Substrate switching, high-affinity substrate uptake, H_2_ utilization; EET and sporulation in specific lineages	Substrate adaptation and H_2_ utilization are supported in SRB; EET and carbon-triggered sporulation remain lineage- or context-dependent mechanisms requiring further validation	Bao et al., 2021; Mafane et al., 2025; Saxena et al., 2025a,b

**Table 2:** Representative acidophilic and acid-tolerant sulfate-reducing bacteria associated with AMD and related mining environment.

Representative taxon/strain	Physiological classification	Reported growth pH range (optimum)	Representative metabolic characteristics	Reported habitat/source	References
*Desulfosporosinus acididurans* M1^T^	Moderately acidophilic SRB	3.8–7.0 (optimum 5.5)	Spore-forming sulfate reducer utilizing H_2_, organic acids, alcohols, and carbohydrates	Acidic sediments from the White River and Tinto River systems	Petzsch et al., 2015; Sánchez-Andrea et al., 2015
*Desulfosporosinus acidiphilus* SJ4^T^	Moderately acidophilic SRB	3.6–5.5 (optimum 5.2)	Spore-forming sulfate reducer utilizing H_2_, lactate, pyruvate, glycerol, glucose, and fructose	Acid mine drainage sediment	Alazard et al., 2010; Valdez-Nuñez et al., 2026
*Desulfosporosinus metallidurans* OL^T^	Acidophilic, metal-resistant SRB	4.0–7.0 (optimum 5.5)	Utilizes H_2_, lactate, formate, glycerol, and carbohydrates; reduces sulfate, sulfite, thiosulfate, nitrate, and fumarate	Microbial mat in a tailings dam at a gold-ore mining site	Panova et al., 2021
*Acididesulfobacillus acetoxydans* INE^T^	Moderately acidophilic SRB	3.8–6.5 (optimum 5.0)	Completely oxidizes acetate and other organic acids to CO_2_ during sulfate reduction; generates alkalinity and exhibits membrane lipid remodeling under acid stress	Enrichment culture derived from acidic sediments of the Tinto River, Spain	Valdez-Nuñez et al., 2026
*Desulfovibrio* sp. strain S4	Acid-tolerant SRB	Sulfate reduction detected at pH ≥ 2.0; growth optimum not established	Maintains sulfate-reducing activity under highly acidic conditions and exhibits elevated metal tolerance	Sediment from an AMD sludge pond, Vietnam	Antsiferov et al., 2017; Park et al., 2022

Physiological classification was assigned according to experimentally determined growth-pH ranges, growth optima, and sulfate-reducing activity. Environmental occurrence alone was not considered sufficient evidence of an acidophilic phenotype. Taxonomic assignments follow the original species descriptions or strain reports wherever possible.

### 2.1 Proton stress under acidic conditions

AMD typically exhibits pH values between 2 to 4, exposing resident SRB to persistent proton stress (Skousen et al., 2017; Valdez-Nuñez et al., 2024). Excess extracellular protons disrupt intracellular pH homeostasis and increase the energetic cost of maintaining proton gradients, thereby reducing the energy available for growth and dissimilatory sulfate reduction (He M. et al., 2025; Saxena et al., 2025a,b). Cultured AMD-associated sulfate reducers encompass both acidophilic (aSRB) and acid-tolerant SRB taxa, whereas neutrophilic SRB detected in AMD are generally considered to persist within locally buffered microniches rather than to actively grow under extremely acidic bulk conditions.

Acidophilic sulfate-reducing bacteria (aSRB) have evolved several strategies to mitigate proton stress, including ion transport regulation, membrane lipid remodeling, and membrane potential control. In the moderately acidophilic sulfate-reducing bacterium *Acididesulfobacillus acetoxydans*, the K^+^-transporting ATPase operon *kdpABC* was significantly upregulated, supporting a strain-specific role for enhanced K^+^ uptake in acid-stress adaptation and potentially in maintaining cytoplasmic pH homeostasis (Egas et al., 2025). Membrane lipid remodeling represents another important adaptation. In *A. acetoxydans*, decreasing pH increased core-lipid saturation and mid-chain methylation. Together with the increased abundance of saturated ether-bound lipids, these changes support reduced membrane proton permeability (Sánchez-Andrea et al., 2022; Egas et al., 2025). Under acetate stress, reduced proportions of iso/anteiso-branched-chain fatty acids and accumulation of acetyl-CoA precursors indicate a coordinated response. This response involves both membrane stabilization and metabolic redirection of acetate toward lipid biosynthesis (Egas et al., 2024). These responses indicate that undissociated organic-acid stress can further modify membrane composition and metabolic allocation in this strain. Cyclopropane-fatty-acid accumulation, increased stress-protein production, and enhanced H^+^-ATPase activity have also been observed in acid-adapted sulfate-reducing consortia (Zhang et al., 2026). However, because these responses were detected in mixed communities, their taxonomic attribution remains uncertain.

Acid adaptation also involves membrane potential regulation. Genomic and transcriptomic analyses have identified multiple Na^+^/H^+^ and K^+^/H^+^ antiporters, together with proton-pumping systems, in aSRB (González-Rosales et al., 2022). Nevertheless, gene presence demonstrates physiological capacity rather than activation under acidic conditions. Physiological and proteomic observations in A. acetoxydans further suggest that membrane-potential control is strain dependent rather than a universally demonstrated aSRB trait (Sánchez-Andrea et al., 2022). Acid-adapted sulfate-reducing consortia have shown increased H^+^-ATPase activity and altered abundance or expression of genes associated with sulfate transport and sulfur metabolism, including cysW/cysP, *dsrB, soxY*, and *soxZ* (Zhang et al., 2026). These patterns suggest coordinated regulation of proton homeostasis and sulfur metabolism, but strain-resolved validation is still required.

Beyond membrane homeostasis, low pH can directly affect core metabolic processes. Acidic conditions can disrupt electron transport and intracellular redox balance, reducing the efficiency of energy conservation during sulfate respiration (Hao et al., 1996; Rabus et al., 2015). Low pH is therefore more appropriately understood to constrain sulfate reduction through integrated effects on redox balance, electron transfer, substrate uptake, and energy conservation. Acidic conditions may also influence the chemical context of sulfate reduction by shifting sulfide speciation toward undissociated H_2_S. At low pH, sulfide predominantly occurs as H_2_S rather than HS^−^, which can reduce sulfide-related inhibition and facilitate sulfide diffusion from the aqueous phase (Thauer et al., 1977; Muyzer and Stams, 2008). However, any potential chemical advantage is generally insufficient to offset the physiological and kinetic constraints imposed by acidic environments. Sulfate-reduction performance under acidic conditions remains strongly influenced by pH, electron-donor availability, sulfide toxicity, microbial acclimation, and community structure (Dong et al., 2023; Valdez-Nuñez et al., 2026).

Acid tolerance varies markedly among SRB taxa. Acidophilic SRB can sustain growth or sulfate reduction at substantially lower pH than acid-tolerant taxa, whereas most neutrophilic SRB exhibit rapid activity loss below approximately pH 4 and are more likely to persist within locally buffered microniches in AMD systems (Sánchez-Andrea et al., 2022). Acid tolerance depends on membrane restructuring, ion homeostasis, metabolic reallocation, and maintenance-energy management rather than on a single universal protective mechanism.

### 2.2 Low-temperature constraints on sulfate reduction

Compared with pH tolerance, metal toxicity, and carbon utilization, the effects of temperature on SRB metabolism have received considerably less attention in AMD bioremediation research. Most physiological parameters and reactor kinetic models for SRB are derived from experiments conducted at 25–35 °C, whereas temperatures below 20 °C remain underrepresented (Neculita et al., 2007). In contrast, in situ AMD temperatures are strongly influenced by climatic and hydrogeological conditions and often remain low in high-latitude, high-altitude, or groundwater-fed mining sites, with some systems approaching freezing conditions (Virpiranta et al., 2022; Karnachuk et al., 2025). This mismatch between laboratory and field conditions suggests that sulfate reduction capacities estimated under mesophilic conditions may overestimate SRB performance in cold AMD environments.

Low temperature constrains sulfate reduction primarily through its effects on membrane transport and enzymatic activity. As temperature decreases, membrane fluidity declines. Reduced membrane fluidity limits the transport of sulfate and organic electron donors and can impair membrane-associated electron transfer processes. Low temperature also slows enzymatic reactions. Although reaction rates generally decline with decreasing temperature, direct enzyme-level characterization of adenylylsulfate reductase (AprAB), dissimilatory sulfite reductase (DsrAB), and other sulfate-reduction enzymes under low-temperature conditions remains limited. Together, reduced membrane transport efficiency and slower catalytic turnover provide a mechanistically plausible explanation for the decline in sulfate-reduction rates at low temperature (Tang et al., 2026).

Microbial adaptation to low temperature has traditionally been interpreted through the framework of homeoviscous adaptation, in which microorganisms modify membrane lipid composition, including fatty acid unsaturation, branching patterns, and polar head groups, to maintain appropriate membrane fluidity (Sinensky, 1974; Collins and Margesin, 2019). However, whether the principles of this framework can be extended to SRB remains unresolved because SRB possess distinct and highly diverse membrane architectures compared with commonly studied bacterial models. Lipidomic analyses of the mesophilic SRB *Desulfatibacillum alkenivorans* revealed an unusual membrane-lipid repertoire containing ether-linked lipids and tetraether cardiolipin (Hopmans et al., 2024), suggesting that SRB may employ membrane-stabilization strategies beyond conventional fatty-acid remodeling. However, these observations were obtained under mesophilic conditions and therefore do not demonstrate cold-induced lipid remodeling. Thus, homeoviscous adaptation provides a useful reference model for investigating cold responses in SRB, but the specific membrane mechanisms underlying SRB cold adaptation remain experimentally unresolved.

Consistent with this evidence gap, genome-based analyses of cold-tolerant SRB enriched from low-temperature AMD groundwater have identified genes associated with cold-shock responses and cell-envelope homeostasis, including *cspA, ompC*, and components of the F-type H^+^-transporting ATPase (Xiao et al., 2025). These genes indicate potential mechanisms of cold adaptation, but their presence demonstrates physiological capacity rather than cold-induced activation or a causal role in tolerance.

Proteogenomic analyses of the filamentous sulfate reducers *Desulfonema limicola* and *Desulfonema magnum* identified numerous stress-response systems, including ClpA/ClpS, RbfA, DegP, and σ24-associated envelope-stress proteins (Schnaars et al., 2021). These findings indicate that at least some SRB possess an extensive repertoire of protein-quality-control and stress-response machinery. Because these datasets were not generated under experimentally imposed cold stress, they identify stress-response systems present in SRB but cannot determine whether these proteins are specifically induced during cold adaptation.

EPS-mediated protection and compatible-solute accumulation are established cold-adaptation strategies in other microorganisms, but their roles in SRB remain unverified and are therefore not considered confirmed mechanisms here.

Despite these constraints, psychrophilic and psychrotolerant SRB can sustain sulfate reduction under near-freezing conditions, indicating that low temperature slows rather than precludes biological activity (Robador et al., 2016). Cold-adapted consortia have demonstrated appreciable sulfate removal in AMD treatment systems, although performance often declines during scale-up because of mass-transfer limitations and weakened microbial interactions (Collins and Margesin, 2019; Xiao et al., 2025). Successful remediation in cold AMD environments therefore depends not only on the intrinsic tolerance of individual SRB but also on the maintenance of functional microbial networks and favorable extracellular microenvironments. Compared with acid adaptation, mechanistic understanding of cold tolerance in SRB remains substantially less developed. Current understanding of cold adaptation in SRB relies primarily on physiological observations and genomic analyses (Xiao et al., 2025). Many mechanistic interpretations are therefore extrapolated from controlled multi-omics studies of other psychrophilic microorganisms, because comparable SRB transcriptomic, proteomic, and lipidomic studies under defined cold stress remain scarce (Koh et al., 2017; Ahn et al., 2021).

### 2.3 Heavy metal toxicity and metabolic inhibition

AMD typically contains high concentrations of metals and metalloids such as Fe, Cu, Zn, Cd, Pb, Cr, As, and U. These elements are primary contributors to AMD toxicity and represent key constraints on SRB activity (Nordstrom et al., 2015; Karnachuk et al., 2025). At the enzymatic level, metal ions can interact with thiol groups, carboxyl groups, or metal cofactor-binding sites within enzymes, altering enzyme conformation and reducing catalytic activity. For example, Cu^2+^ has been shown to inhibit hydrogenase activity in *Desulfovibrio* spp., thereby interfering with H_2_-dependent sulfate reduction (Utgikar et al., 2002; Vignais and Billoud, 2007).

AprAB and DsrAB contain redox-active cofactors and are integrated into coordinated electron-transfer networks that support sulfate reduction (Grein et al., 2013). Although Fe–S-containing proteins are generally vulnerable to metal displacement and oxidative damage, direct SRB-specific evidence demonstrating metal-induced structural disruption of AprAB- or DsrAB-associated cofactors remains limited. Toxic effects also differ among metals; Cu(II), for example, may cause strong cellular toxicity, whereas Zn(II) can exert pronounced inhibition of sulfate-reducing activity in surviving populations (Utgikar et al., 2003). Thus, evidence for metal inhibition of sulfate reduction is currently stronger at the whole-cell and hydrogenase activity levels than at the level of direct structural disruption of AprAB or DsrAB.

Beyond direct enzyme inhibition, heavy metals can promote oxidative stress and damage DNA, proteins, and membranes (Jang and Imlay, 2007). Although much of the canonical understanding of metal-induced reactive oxygen species derives from non-SRB model organisms, SRB-specific omics studies demonstrate that metal exposure triggers broad responses involving energy metabolism, transport, and stress regulation.

In *Desulfovibrio vulgaris*, exposure to Cu(II) or Hg(II) prolonged the lag phase and increased transcripts encoding ATP-binding proteins and ATPase-related functions, indicating an increased energetic cost of metal adaptation (Chang et al., 2004). Protein-interaction analyses under mercury exposure further indicate that resistance pathways form an early response accompanied by perturbations in carbon, nitrogen, and sulfur metabolism (Dong et al., 2024a).

Metal ions can also compromise membrane integrity, disrupting nutrient uptake, waste-product export, and membrane-associated electron transport. Accumulation of metal-sulfide precipitates on cell surfaces may additionally form a physical barrier that limits substrate access (Villa-Gomez et al., 2014).

To mitigate heavy metal-induced cytotoxicity, some SRB employ extracellular defense mechanisms in which extracellular polymeric substances (EPS) bind metal ions and facilitate localized metal sulfide precipitation, thereby reducing metal bioavailability and limiting diffusion of toxic metals to the cell envelope (Xu and Chen, 2020; Gan et al., 2023; Huang et al., 2023). The relative contributions of passive adsorption, active EPS production, and mineral nucleation are likely to vary with metal identity, pH, sulfide concentration, and community composition. Accordingly, EPS binding and localized precipitation are supported in SRB-containing systems, whereas a strict temporal separation into acute and chronic defense phases remains hypothetical.

Intracellular metal homeostasis may additionally involve active transport. Quantitative proteomic analysis of *Desulfovibrio desulfuricans* ND132 identified multiple metal-transport proteins, including putative heavy-metal and Cu-export systems, demonstrating expressed efflux capacity in at least some SRB (Qian et al., 2018). In *Desulfovibrio alaskensis* exposed to Pt and Pd, shotgun proteomics further revealed coordinated changes in metal transport, reduction, and broader stress-response pathways. Changes in a [NiFe]-hydrogenase subunit also affected nanoparticle formation, indicating strain-specific involvement of hydrogenases in metal reduction (Capeness et al., 2019).

These findings support roles for energy-dependent transport and metal homeostasis but do not establish resistance–nodulation–division (RND) pumps as the dominant or universal resistance mechanism in SRB. RND-mediated export should therefore be regarded as a plausible, strain-specific mechanism rather than a universally validated SRB response.

Sulfide generated through dissimilatory sulfate reduction can bind with metal ions to form low-solubility metal sulfide precipitates, such as FeS, CuS, and ZnS. In simulated tailings–AMD contamination systems, SRB can immobilize Pb, Zn, Fe, Cu, and Cr, through sulfide precipitation, biosorption, and interactions in mixed microbial communities. These processes reduce dissolved-metal concentrations, mobility, and bioavailability (Dong et al., 2024b).

Accordingly, sulfide precipitation currently has the strongest direct experimental support among the metal-mitigation mechanisms discussed here. EPS-mediated protection is also supported in SRB-containing systems, whereas the roles of specific efflux systems and direct protection of Fe–S-containing enzymes remain less well validated. When metal concentrations exceed physiological tolerance thresholds, oxidative stress, membrane dysfunction, impaired transport, and enzyme inhibition converge to suppress sulfate reduction. Thus, although SRB-derived sulfide contributes to metal immobilization, heavy metals remain major constraints on SRB activity.

### 2.4 Carbon and electron-donor limitation

Many AMD environments, particularly mine groundwater or oligotrophic pit lakes, lack sufficient biodegradable organic carbon (Jamil and Clarke, 2013; Bagheri Novair et al., 2024). Therefore, electron donor scarcity directly reduces SRB growth rates, biomass yields, and sulfate reduction rates, thereby weakening metal sulfide precipitation and acidity neutralization.

SRB exhibit substantial diversity in substrate-utilization spectra. *Desulfovibrio* spp. commonly utilize lactate, pyruvate, ethanol, formate, or H_2_; *Desulfobacter* spp. can completely oxidize acetate; and members of *Desulfococcus* can utilize long-chain fatty acids (Bagheri Novair et al., 2024). Where carbon sources are heterogeneous or scarce, community-level complementarity may broaden the range of substrates available to sulfate reducers, although this benefit depends on fermentative partners, transport conditions, and competition with other anaerobic guilds. Some SRB possess transport systems that operate effectively at low substrate concentrations. Transcriptomic evidence demonstrates condition-specific transporter regulation and carbon-flow reprogramming under resource limitation. Under lactate limitation, *Desulfobacterium* autotrophicum increased complete lactate oxidation through the Wood–Ljungdahl pathway. Under sulfate limitation, it shifted toward incomplete substrate oxidation and differentially expressed sulfate transporters with distinct predicted affinities (Marietou et al., 2022). These findings support condition-dependent transporter regulation and metabolic rerouting in at least one SRB model.

When organic carbon is severely depleted, SRB capable of using inorganic electron donors may become important for maintaining sulfate reduction. Many SRB encode [NiFe] hydrogenases, and some taxa also harbor [FeFe] hydrogenases, enabling H_2_-supported dissimilatory sulfate reduction (Sreekutty and Chellapandi, 2024). In AMD environments, H_2_ may be generated through fermentation, corrosion of reactive metals, and water–rock interactions (Karnachuk et al., 2025). However, the magnitude, spatial distribution, and continuity of H_2_ supply must be established through direct geochemical measurements in individual AMD systems. Some SRB also exhibit extracellular electron-transfer capabilities. Electrochemical and proteomic studies show that particular Desulfovibrio strains can exchange electrons with electrodes, minerals, or conductive surfaces (Valdez-Nuñez et al., 2022; Liu et al., 2024). Transcriptomic and proteomic analyses of D. vulgaris biofilms have further revealed increased abundance of Ech hydrogenase, formate dehydrogenase, and Rnf-type oxidoreductase components, indicating reorganization of hydrogen, formate, and membrane-associated electron-transfer pathways during surface-associated growth (Clark et al., 2012). These findings demonstrate extracellular electron transfer in selected SRB–surface systems, but do not establish conductive minerals as a significant electron source for sulfate reduction in natural AMD environments. This proposed mechanism therefore remains hypothetical under AMD conditions.

When electron-donor supply falls below the cellular maintenance-energy threshold, some spore-forming SRB may enter dormant states. Members of *Desulfosporosinus* and *Desulfotomaculum* can sporulate, and their spores may persist through unfavorable periods before germinating when suitable conditions return. The frequent detection of *Desulfosporosinus* in acidic mining environments is consistent with a role for sporulation in lineage persistence (Alazard et al., 2010). However, direct evidence that carbon depletion specifically triggers sporulation in AMD or that dormant spores subsequently re-establish in situ sulfate reduction remains limited. Sporulation should therefore be regarded as a taxon-restricted persistence strategy rather than a universal SRB response to electron-donor limitation.

Overall, direct evidence links electron-donor limitation to reduced sulfate reduction, transporter regulation, metabolic rerouting, and increased reliance on H_2_. Mineral-mediated electron transfer and carbon-triggered sporulation remain context dependent or insufficiently validated.

### 2.5 Metabolic niche contraction under multiple stressors

The following discussion integrates observations from SRB physiology and general microbial stress biology and should be regarded as a conceptual interpretation rather than a fully validated mechanistic model.

**Figure 1:**
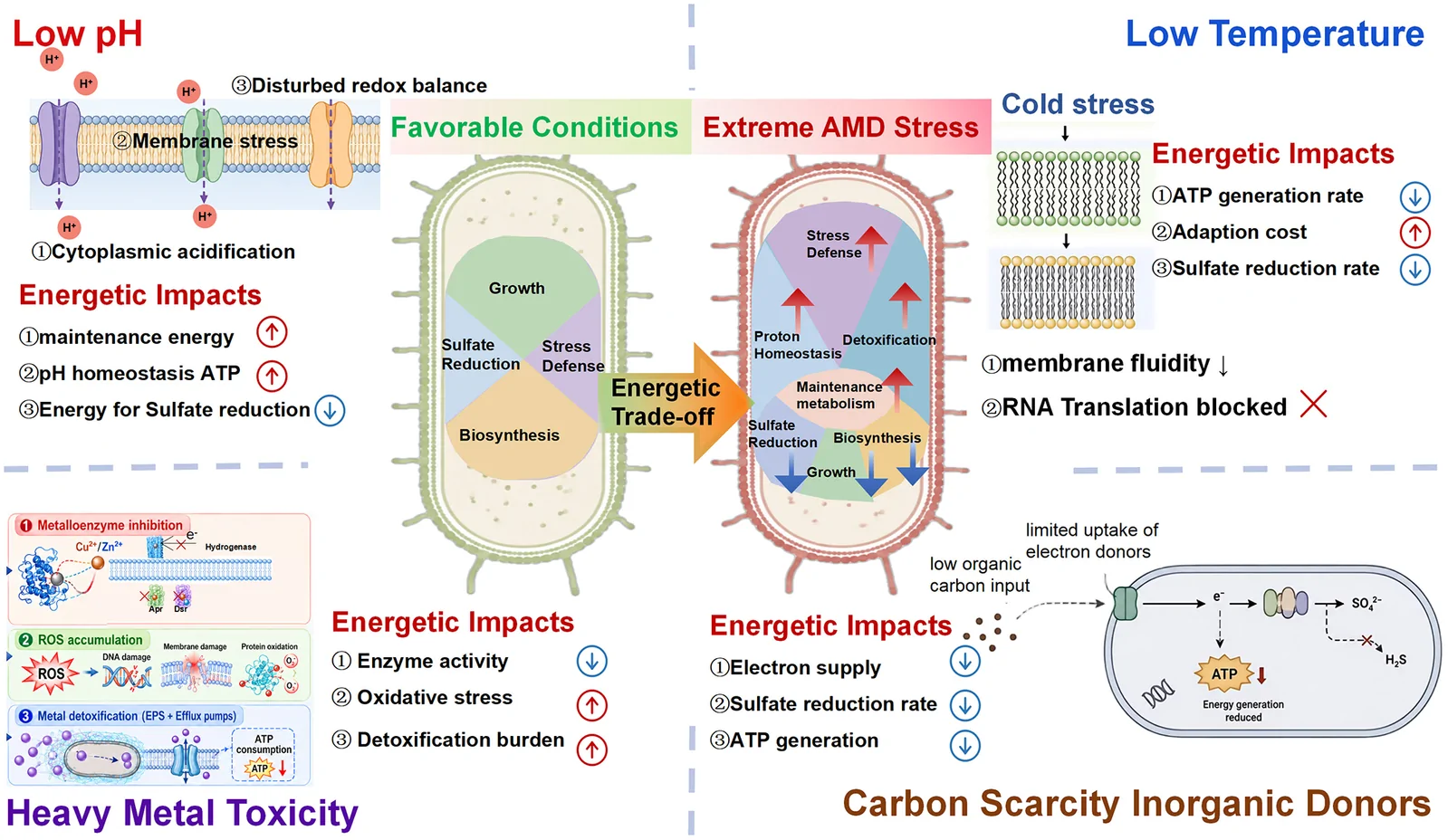
Energetic trade-offs and resource allocation shifts in SRB under multiple AMD stressors. Under favorable conditions (center-left), SRB balance energy among growth, biosynthesis, and sulfate reduction. Exposure to key AMD constraints—low pH, low temperature, heavy metal toxicity, and carbon scarcity (outer quadrants)—forces a metabolic redirection toward cellular maintenance and defense (center-right), ultimately contracting the metabolic niche of SRB.

In natural AMD waters, low pH, low temperature, heavy metal toxicity, and organic carbon scarcity often coexist and interact. Proton extrusion, metal detoxification, protein repair, and membrane stabilization increase non-growth-associated energy expenditure. Simultaneously, low temperature reduces ATP-generation rates, whereas electron-donor limitation restricts the total energy available from substrate oxidation. Multiple stressors may progressively reduce the effective metabolic niche of SRB by simultaneously increasing maintenance costs and limiting energy acquisition (Tijhuis et al., 1993). As summarized in Figure 1, increasing maintenance energy expenditure progressively reduces the energy available for biomass production and sulfate reduction and may eventually make growth energetically or kinetically unsustainable (Lever et al., 2015)

Direct multi-omics studies simultaneously examining the combined effects of major AMD stressors on SRB remain rare. Genome-resolved metatranscriptomics analyses of peatland SRB communities showed that *dsrAB* transcripts remained among the most abundant under anoxic conditions, whereas genome-wide transcription declined during oxic phases without complete transcriptional shutdown. Energy-metabolism and oxygen-defense genes continued to be expressed, suggesting that SRB maintain core metabolic functions during transient oxygen exposure (Dyksma and Pester, 2024). Although this was not an AMD system, it illustrates that SRB persistence under recurrent stress can involve reversible metabolic downregulation rather than immediate functional extinction.

At the membrane level, interacting stressors may impose opposing physical requirements. Acid adaptation generally favors reduced proton permeability through decreased membrane permeability, whereas low-temperature adaptation requires sufficient membrane fluidity to maintain membrane protein function and electron transport (Sinensky, 1974; Lund et al., 2014). Together, these contrasting requirements suggest a potential trade-off between proton protection and membrane fluidity during combined acid and cold stress. However, this hypothesis has not yet been directly evaluated in sulfate-reducing bacteria using combined acid–temperature lipidomic or membrane biophysical analyses.

The interaction among multiple AMD stressors can generate effects that are not predictable from single-factor experiments. For example, low pH combined with carbon limitation may intensify organic-acid stress when partially oxidized fermentation products accumulate. Low temperature may further aggravate metal stress by limiting detoxification, cellular repair, and other protective responses. In addition, the toxicity of mixed metals may deviate from additive effects, with some combinations producing synergistic inhibition and others showing antagonistic interactions (Nielsen et al., 2018). Consequently, tolerance thresholds derived from single-stressor experiments may overestimate SRB performance under authentic AMD conditions.

As environmental constraints intensify, increased maintenance-energy requirements may reduce the competitive capacity of SRB relative to microbial populations better adapted to specific combinations of acidity, metal exposure, and carbon limitation (Lever et al., 2015; Valdez-Nuñez et al., 2024). Thus, multistressor conditions influence not only SRB physiology but also the microbial interactions that support sulfate reduction.

These interactions help explain the recurrent discrepancy between laboratory and field-scale AMD remediation. Under controlled laboratory conditions, SRB often exhibit high sulfate-reduction efficiency, whereas field systems usually experience lower and more variable performance due to fluctuating stress conditions, transport limitations, spatial heterogeneity, and dynamic microbial interactions (Johnson and Quatrini, 2020). Under such conditions, SRB may become increasingly dependent on community-level processes, including electron-donor supply, redox regulation, and the formation of protected microenvironments, to sustain sulfate reduction.

This dependence provides a link between physiological stress responses and the community-level interactions discussed in the following section

Genome-resolved multi-omics studies have provided important insights into the genetic basis of sulfate reduction and stress adaptation in SRB. Genes encoding key sulfate-reduction enzymes (e.g., *sat, aprAB*, and *dsrABD*) and associated electron-transfer complexes such as *qmoABC* and *dsrMKJOP* have been identified, providing information on the potential energy-conservation pathways of sulfate-reducing bacteria (Pereira et al., 2011; Dörries et al., 2016). Comparative genomic analyses have further revealed genes associated with oxidative defense, molecular chaperones, metal homeostasis, and other stress-response functions (Zhou et al., 2011). However, these genomic inventories primarily indicate physiological potential rather than active responses under environmental conditions. Strain-resolved metatranscriptomic, metaproteomic, lipidomic, and metabolomic evidence from authentic AMD environments remains scarce, limiting direct validation of mechanisms inferred from laboratory studies.

Although physiological tolerance determines whether SRB survive, sulfate reduction in AMD is ultimately realized within microbial communities. Future experiments should therefore employ diffusion-restricted, multispecies, and long-term experimental designs that better reproduce the physicochemical and ecological heterogeneity of AMD systems while explicitly manipulating combinations of acidity, metals, temperature, and electron-donor availability (Neculita et al., 2007; Ayangbenro et al., 2018; Rambabu et al., 2020). Integrating these factorial designs with strain-resolved multi-omics approaches, including transcriptomics, proteomics, lipidomics, and metabolomics, will help identify the environmental conditions supporting sustained SRB activity and distinguish active stress responses from latent genetic potential or mechanisms inferred from non-SRB microorganisms (Zhou et al., 2011; Hopmans et al., 2024).

## 3 Ecological interactions and functional constraints shaping SRB activity in AMD microbial communities

Although the preceding sections focused on how environmental stress constrains the metabolic niche of individual SRB, sustained sulfate reduction in AMD is strongly influenced by community-level interactions. Multiple stressors simultaneously increase cellular maintenance requirements and reduce energy-generating capacity, leaving less energy available for growth and sulfate reduction. As this energy margin declines, SRB may become increasingly dependent on associated microbial populations for substrate supply, redox stabilization, and protection from environmental stress. Community stability can therefore be considered from an ecological resilience perspective, where functional persistence depends on the ability of microbial communities to maintain or adjust interactions under environmental disturbances.

**Figure 2:**
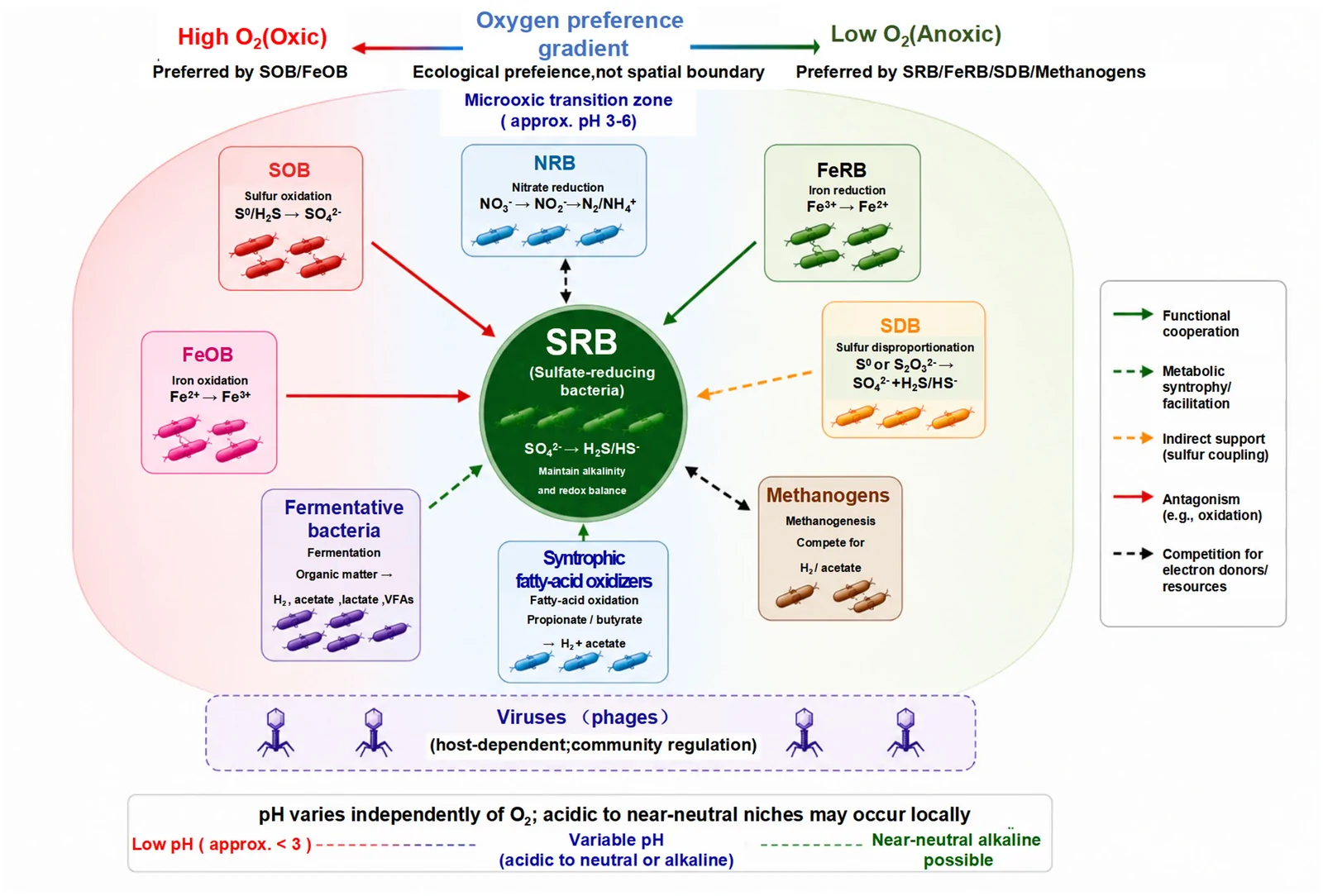
Microbial network regulation of SRB function in AMD ecosystems. The figure illustrates how SRB-mediated sulfate reduction is shaped by both supportive and antagonistic microbial interactions along oxygen and redox gradients. Fermentative, syntrophic, iron-reducing, and habitat-engineering microorganisms support SRB by maintaining electron donor supply, redox balance, and local niche stability, whereas sulfur-oxidizing, iron-oxidizing, nitrate-reducing microorganisms and methanogens may restrict SRB activity through acidification, sulfide re-oxidation, or electron donor competition. The figure emphasizes that SRB persistence depends on network-level stability rather than SRB abundance alone.

Major transformations of carbon, sulfur, iron, and nitrogen are distributed among multiple microbial guilds rather than encoded within a single taxon. Genome-resolved multi-omics studies accordingly show that elemental transformations in AMD are organized into interconnected community-scale networks (Ayala-Muñoz et al., 2022; Luo et al., 2023). As summarized in Figure 2, these networks involve both cooperative and competitive interactions among functional guilds that collectively regulate sulfate-reduction efficiency and long-term persistence under AMD conditions. Fermentative microorganisms, syntrophic partners, FeRB, SDB, and habitat-engineering populations may promote sulfate reduction by sustaining electron-donor delivery and buffered reductive niches, whereas SOB, FeOB, NRB, methanogens, and other populations may constrain it through substrate competition, electron diversion, acid generation, redox modification, and sulfide reoxidation. These relationships are context-dependent and often represent coupled biogeochemical processes across spatially differentiated niches rather than uniformly cooperative or antagonistic interactions. The following sections examine how these interactions regulate three proximate determinants of sulfate-reduction persistence: electron-donor delivery, electron-flux partitioning, and the maintenance of buffered redox microenvironments. A detailed comparison of the relevant functional guilds is provided in Supplementary Table S1.

### 3.1 Syntrophic metabolic networks regulating electron flow and constraining SRB activity

The scarcity and limited bioavailability of dissolved organic carbon represent major ecological bottlenecks for SRB activity in AMD environments. In the absence of sustained external carbon inputs, SRB frequently depend on fermentation-centered syntrophic networks for electron-donor acquisition. Fermentative microorganisms degrade complex organic matter into H_2_, formate, lactate, acetate, and other volatile fatty acids (VFAs), which can subsequently be consumed by SRB. The continuous removal of these products maintains low end-product concentrations and improves the thermodynamic feasibility of further substrate degradation, thereby creating a tightly coupled syntrophic network. Substrate use nevertheless differs among SRB lineages: incomplete oxidizers preferentially use H_2_, formate, and lactate, whereas complete oxidizers can additionally metabolize organic acids such as acetate (Wang D. et al., 2025). Consequently, fermenter–SRB interactions involve not only organic-acid exchange but also interspecies hydrogen transfer (IHT) and interspecies formate transfer (IFT), with H_2_ serving as an especially important electron donor in many AMD enrichment systems. In addition to IHT and IFT, extracellular electron transfer may provide a supplementary route for electron exchange between some SRB, minerals, and syntrophic partners, although its quantitative importance in AMD communities remains less well resolved (Zeng et al., 2019; Harshita et al., 2026).

IHT and IFT provide a thermodynamic foundation for this syntrophic organization. Continuous consumption of H_2_ or formate by SRB creates a thermodynamic pull that facilitates otherwise unfavorable oxidation reactions performed by fermentative partners (Thauer et al., 1977). This coupling can produce marked metabolic specialization among functional groups. In low-temperature AMD enrichment systems, for example, glycerol was sequentially converted by primary fermenters, including *Cellulomonas* and *Anaerovorax*, secondary fermenters such as *Desulfitobacterium*, and H_2_-utilizing sulfate reducers represented by *Desulfosporosinus*, forming a three-tier syntrophic metabolic chain (Dev et al., 2021). Comparable functional organization has been observed at field scale, suggesting that interactions among hydrolytic, fermentative, and sulfate-reducing populations may be conserved despite substantial variation in taxonomic composition (Jurado et al., 2025).

SRB are not merely passive recipients of fermentation products but can function as metabolically flexible participants within these networks. When sulfate is temporarily depleted or respiratory conditions become unfavorable, some SRB shift from sulfate respiration to fermentative or syntrophic metabolism, converting substrates such as pyruvate and lactate into H_2_, formate, acetate, ethanol, and other products. When sulfate becomes available again, they can resume sulfate reduction and consume low-molecular-weight electron donors (Plugge et al., 2011). Such reversible metabolic switching may enhance persistence in AMD environments, where steep microscale gradients of sulfate, pH, oxygen, and electron-donor availability are common. Studies of Desulfovibrio gigas further showed that hydrogenases can mediate both H_2_ uptake during sulfate respiration and H_2_ production during fermentation, reinforcing the view that SRB dynamically alternate between different metabolic roles according to local environmental conditions (Morais-Silva et al., 2013).

In addition to classical fermentative bacteria, genome-resolved studies have identified previously underappreciated microbial groups that may contribute to syntrophic carbon turnover. Candidate Phyla Radiation (CPR) bacteria and DPANN archaea have been detected in some AMD sediments and are predicted to possess metabolic capacities potentially involved in complex organic-matter degradation and acetate-producing fermentation pathways (Peng S.-X. et al., 2025). These metabolically specialized lineages may indirectly influence SRB activity by expanding carbon transformation pathways and increasing the availability of intermediate metabolites. However, their specific contributions to sulfate reduction remain largely inferred from genome-based predictions rather than experimentally validated interactions (He Y. et al., 2025). More broadly, genome-resolved metagenomic reconstruction indicates that AMD syntrophy extends beyond pairwise fermenter–SRB interactions. Complementary functions involving carbon degradation, nitrogen transformation, sulfur metabolism, and iron cycling are distributed among phylogenetically diverse populations that collectively support electron transfer and carbon turnover within community-wide networks (Luo et al., 2023).

Despite their importance, syntrophic networks are constrained by both competition for metabolites and the accumulation of inhibitory intermediates. Methanogenic archaea can compete directly with SRB for H_2_ and acetate (Muyzer and Stams, 2008; Stams and Plugge, 2009). Under sulfate-replete conditions, sulfate reduction is generally thermodynamically favored over methanogenesis, which can give SRB a competitive advantage for shared electron donors (Jackson and McInerney, 2002). Carbon limitation may intensify this competition by restricting substrate availability, although methanogenic activity is usually strongly suppressed under the highly acidic conditions typical of AMD. At the same time, incomplete consumption of fermentation products can destabilize the network. Acetate is an important intermediate for fermenters and some SRB, but under acidic conditions its undissociated form can diffuse across cell membranes, dissociate intracellularly, and disrupt pH homeostasis (Russell and Francisco, 1997). In the acidophilic SRB *Acididesulfobacillus acetoxydans*, acetate stress inhibited growth and induced extensive metabolic remodeling (Egas et al., 2024), while acetate accumulation in low-temperature AMD enrichment systems was associated with a marked decline in sulfate-reduction efficiency (Virpiranta et al., 2022). Thus, fermentation supplies the electron donors required for sulfate reduction, but inadequate product removal can cause the same intermediates to become inhibitory.

Taken together, these observations indicate that sulfate reduction in AMD may be constrained less by the mere presence of sulfate-reduction genes than by the quantity, quality, and spatial delivery of organic carbon to anoxic niches, as well as by local redox conditions that favor sulfur reoxidation. This interpretation is consistent with depth-resolved metagenomic analyses of a permanently stratified acidic pit lake, in which the realized activity of sulfate-reducing communities depended strongly on carbon availability and redox zonation rather than on genetic potential alone (Ayala-Muñoz et al., 2022).

### 3.2 Redox interactions between iron–sulfur cycling functional guilds and SRB

Iron and sulfur cycling in AMD is jointly mediated by SOB, FeOB, FeRB, SDB, and SRB. The functional partitioning, competition, and metabolic coupling among these guilds along redox gradients influence acid–base evolution, secondary mineral formation, contaminant mobility, and the ecological stability of SRB. Because iron and sulfur transformations are tightly interconnected, these microbial interactions also shape the long-term performance and evolutionary trajectory of AMD bioremediation systems (Whaley-Martin et al., 2023; Zhou et al., 2025).

At the oxidative end of the redox gradient, SOB and FeOB serve not only as the primary biological drivers of AMD generation and persistence but also as the most direct biological antagonists of SRB-mediated remediation products. Long-term multi-omics investigations have demonstrated that SOB dynamically shift their metabolic strategies in response to redox conditions. Under oxic conditions, sulfur oxidation is primarily mediated through the complete Sox pathway, whereas under oxygen-limited conditions, SOB increasingly rely on incomplete Sox and reverse dissimilatory sulfite reductase (rDSR) pathways (Whaley-Martin et al., 2023). The acid-generating capacity of SOB is therefore not fixed but represents a redox-dependent metabolic phenotype. Changes in oxygen penetration or partial pressure can consequently regulate net sulfur oxidation and acidification, thereby altering the suitability of local niches for SRB.

This oxidative pressure is reinforced under strongly acidic conditions, where typical FeOB and SOB taxa such as Acidithiobacillus and Leptospirillum commonly become dominant while SRB and their syntrophic partners decline (Chen et al., 2015). Sustained iron and sulfur oxidation lowers pH and increases the concentrations of oxidized iron and other toxic metals, directly inhibiting SRB physiology. Reduced SRB activity in turn decreases sulfide production and alkalinity generation, weakening biological mechanisms that might otherwise counteract acidification. These processes can form a self-reinforcing feedback loop in which acid generation suppresses reductive guilds, reduced reductive activity further limits neutralization, and the system becomes stabilized in an acidic, oxidative state dominated by SOB and FeOB.

Toward the reductive end of the gradient, interactions between FeRB and SRB may shift from competition or indirect antagonism to temporal cooperation. In coupled schwertmannite-reduction experiments involving *Shewanella oneidensis* MR-1 and *Desulfosporosinus meridiei*, FeRB initially reduced structural Fe(III), releasing mineral-bound sulfate and promoting vivianite formation. SRB subsequently used the liberated sulfate, leading to the formation of mackinawite and siderite (Ke C. et al., 2023). SRB-derived H_2_S can also chemically reduce Fe(III) under acidic conditions, producing Fe^2+^ and reduced iron–sulfur minerals. SRB therefore contribute to iron reduction not only through ecological interactions with FeRB but also through abiotic reactions driven by their metabolic products (Becerra et al., 2024).

This temporally coupled process can be interpreted as a form of metabolic facilitation or asymmetric cooperation. FeRB increase sulfate availability by mobilizing mineral-bound sulfate, while sulfide produced by SRB reacts with Fe^2+^ to form FeS. Sulfide precipitation may alleviate inhibition caused by the accumulation of dissolved H_2_S and thereby support continued sulfate reduction. A non-competitive sulfide inhibition constant of approximately 251 mg L^−1^ (as S) has been reported for *Desulfovibrio desulfuricans* (Reis et al., 1992), suggesting that efficient FeS precipitation could partially protect SRB at elevated sulfide concentrations. However, stronger SRB activity or greater sulfide production should not automatically be interpreted as improved remediation. FeRB–SRB-driven transformations can alter mineral structure, sorption capacity, and contaminant partitioning, and their net effect depends on the specific mineral transformation pathway and prevailing geochemical boundary conditions (Gao et al., 2024).

In addition to FeRB, sulfur-disproportionating microorganisms provide an alternative reductive pathway linking oxidized and reduced zones. Elemental sulfur (S^0^) and thiosulfate (S_2_${\text{O}}_{3}^{2-}$), which can accumulate during incomplete sulfur oxidation, are not necessarily terminal products. In carbon-limited reductive zones of AMD systems, these sulfur intermediates can be utilized by sulfur-disproportionating bacteria (SDB), which generate H_2_S and S${\text{O}}_{4}^{2-}$ through chemolithoautotrophic sulfur disproportionation. Recent studies have shown that SDB can sustain stable sulfide production even in the absence of external organic carbon inputs, highlighting the strong compatibility between their metabolic requirements and the characteristic “high-sulfate, low-organic-carbon” conditions of AMD environments (Zou et al., 2023). Unlike heterotrophic SRB, SDB do not necessarily depend on organic electron donors and may therefore maintain localized sulfidic conditions when fermentative substrate supply is insufficient. Their H_2_S can precipitate dissolved metals as metal sulfides and potentially reduce metal bioavailability within nearby SRB microhabitats. Sulfur disproportionation should therefore be regarded as a complementary sulfide-generating process that connects oxidative sulfur transformations with reductive metal immobilization, while remaining mechanistically distinct from canonical sulfate reduction.

Across the full redox gradient, oxygen penetration depth determines the relative spatial extent of the SOB/FeOB-dominated oxidative zone and the FeRB/SRB-dominated reductive zone, making hydrological and oxygen control practical engineering targets in passive AMD treatment systems. Long-term system stability, however, also depends on the mineralogical evolution of the products generated by these interactions. Mackinawite (FeS), a common initial precipitate, is thermodynamically metastable and may require months to years to transform into more stable phases such as pyrite (FeS_2_) (Rickard and Luther, 2007). During this transitional period, mackinawite remains vulnerable to oxidative disturbance. Hydrological fluctuations that introduce oxygen can promote rapid reoxidation of metal sulfides, potentially causing pulse releases of previously immobilized metals. The long-term effectiveness of SRB-based treatment therefore depends not only on short-term sulfate reduction and alkalinity generation but also on mineral aging, hydrological stability, and resistance to reoxidation.

Depth-resolved metagenomic and metatranscriptomic analyses of a permanently stratified acidic pit lake demonstrated that oxidative and reductive microbial processes are spatially organized along vertical redox gradients, with iron oxidation predominating in oxic waters, whereas sulfate reduction, iron reduction, and sulfur cycling become increasingly important across transition and anoxic zones (Ayala-Muñoz et al., 2022). Functional coupling among these guilds does not require direct physical coexistence. Dissolved sulfur intermediates, iron species, organic compounds, and electron carriers can move across redox interfaces, enabling indirect interactions among microorganisms occupying distinct microenvironments. Ecological relationships among SOB, FeOB, FeRB, SDB, and SRB should therefore be interpreted as community-scale biogeochemical coupling across spatially stratified redox niches rather than solely as direct cell-to-cell associations.

### 3.3 Interactions between nitrate-reducing bacteria and SRB in coupled nitrogen–sulfur cycling

Nitrogen and sulfur biogeochemical cycles in AMD are tightly coupled through competition for electron donors, metabolite exchange, and redox gradients. Recent evidence indicates that some SRB possess metabolic capacities beyond canonical sulfate reduction. For example, the acidophilic SRB *Acididesulfobacillus acetoxydans* employs the NADH-dependent sulfite reductase AsrABC for both sulfite and nitrite reduction, directly linking sulfate reduction with the dissimilatory nitrate reduction to ammonium (DNRA) pathway (Egas et al., 2024). This enzymatic bifunctionality suggests that some AMD-associated SRB can contribute simultaneously to sulfur and nitrogen transformations. A comparable coupling between sulfur disproportionation and DNRA has been reported in Desulfurivibrio dismutans from alkaline lakes, indicating that integrated sulfur–nitrogen metabolism may occur across taxonomically and geochemically distinct ecosystems (Sorokin et al., 2025).

SRB may also contribute to nitrogen acquisition capacity in AMD systems. In low-oxygen AMD environments, SRB-enriched communities may harbor nitrogen-fixing potential, and coordinated increases in the abundance of *nifH* and *dsrAB* suggest a potential association between nitrogen acquisition and sulfate reduction (Liu et al., 2024). Although gene abundance alone does not demonstrate direct physiological coupling at the cellular level, nitrogen fixation could reduce dependence on externally supplied fixed nitrogen and potentially enhance the persistence of SRB-centered communities. Recent studies of coupled Fe–S–N biogeochemical processes have demonstrated close interactions among ammonium oxidation, Fe(III) reduction, and sulfate reduction, suggesting that nitrogen transformations are integrated with iron and sulfur redox cycling rather than occurring as isolated processes (Ye et al., 2025).

At the community level, relationships between NRB and SRB include both competition and niche differentiation. Co-occurrence networks in AMD-contaminated soils show weakened positive associations and stronger negative correlations between NRB and SRB, consistent with competition for shared electron donors or occupation of contrasting redox niches. Nevertheless, some sulfate-reducing taxa may respond positively to nitrogen availability. *Desulfobacca*, for example, was positively associated with nitrate and functioned as a network hub in AMD-contaminated soil communities (Dong et al., 2024b). Such patterns suggest that NRB–SRB coexistence may be maintained through spatiotemporal niche partitioning and taxon-specific metabolic responses rather than through uniform competitive exclusion.

NRB can additionally suppress sulfate reduction through the production of reactive nitrogen intermediates. In nitrate-mediated sulfide-control systems, nitrite generated by NRB promotes oxidation of SRB-derived sulfide and alters local redox conditions, thereby reducing SRB activity. NRB-derived biosurfactants can further enhance nitrate utilization and prolong the inhibitory effect (Fan et al., 2020). Because these mechanisms have been demonstrated mainly under circumneutral conditions, their quantitative importance in strongly acidic AMD environments remains uncertain. Nevertheless, they illustrate a broader ecological mechanism through which NRB can regulate SRB niche occupancy by modifying sulfide availability and local redox chemistry.

Overall, NRB–SRB relationships in AMD are unlikely to be uniformly cooperative or antagonistic. They instead reflect a dynamic balance among electron-donor competition, sulfur and nitrogen metabolite exchange, redox partitioning, enzymatic multifunctionality, and functional redundancy. Through these interactions, SRB may act not only as drivers of sulfur cycling but also as components of integrated nitrogen–sulfur–iron networks that enhance community adaptation in oligotrophic and chemically extreme environments.

### 3.4 Self-organized habitat engineering and resilience boundaries of AMD interaction networks

The stability of SRB-driven sulfate reduction networks in AMD depends on the sustained persistence of interacting microbial populations under extreme physicochemical conditions. At the cellular level, stress-response mechanisms provide the physiological basis for SRB survival under unfavorable conditions. In *Acididesulfobacillus acetoxydans*, acetate exposure (7.5 mM) induces downregulation of glutamate and aspartate biosynthesis, membrane lipid remodeling, and redistribution of fatty acid precursors, which together contribute to reduced proton permeability and altered energy allocation. These adjustments allow sulfate-reducing activity to persist under acetate stress while reflecting a trade-off between stress tolerance and metabolic efficiency (Egas et al., 2024). Beyond individual-cell responses, long-term network stability relies on interactions among microbial populations. In acidophilic SRB communities, *Desulfosporosinus* and *Acididesulfobacillus* frequently co-dominate and exhibit complementary substrate utilization patterns involving glycerol and acetate. Such functional complementarity may reduce intermediate metabolite accumulation and support community stability under low-pH conditions through coordinated carbon transfer and electron-flow regulation (Valdez-Nuñez et al., 2026).

Beyond metabolic complementarity, AMD microbial communities can actively engineer local habitats that extend the realized niche of SRB. Acidophilic heterotrophs and phototrophs capable of biofilm formation and extracellular polymeric substance (EPS) secretion create physicochemical barriers around cells and mineral surfaces. Functional groups within EPS, including carboxyl, hydroxyl, phosphate, and amino groups, can bind Fe, Al, Cu, and Zn, reducing their bioavailability. Dense biofilm matrices can also limit oxygen and proton diffusion, generating microscale pH and redox buffering (Dong et al., 2024b). Microalgal EPS similarly adsorb heavy metals via extracellular complexation, mitigating local metal stress (Momin et al., 2024). Ureolytic bacteria release N${\text{H}}_{4}^{+}$ and HCO_3_- through urea hydrolysis, neutralizing acidity and promoting metal carbonate precipitation, thereby facilitating SRB colonization (Peng C. et al., 2025; Ren et al., 2025). Although these organisms do not directly reduce sulfate, their combined effects on acidity, metal bioavailability, and spatial shielding can create ecological refuges for SRB and their syntrophic partners.

Habitat engineering further generates spatial heterogeneity. Microscale oxygen and redox gradients within biofilms and mineral aggregates allow SRB to persist in anoxic microniches and retain sulfate-reducing activity under macroscopically oxic conditions (Ito et al., 2002; Bryukhanov et al., 2010). Such microscale separation can support the coexistence of iron-oxidizing, sulfur-oxidizing, fermentative, iron-reducing, and sulfate-reducing processes within the same macroscopic habitat. However, the protective effects of EPS, biofilms, carbonate precipitation, and metabolic cooperation remain finite and decline when environmental stress exceeds the combined buffering capacity of the community.

Viral and eukaryotic components may represent additional, although currently less understood, factors influencing AMD microbial networks. Metagenomic surveys have identified abundant viral operational taxonomic units (vOTUs) whose distributions are structured by pH, redox potential, metal load, and host-community composition (Gao et al., 2022; Hu et al., 2025). Auxiliary metabolic genes (AMGs), particularly those associated with carbon and sulfur cycling, have been detected in AMD viromes and may be associated with host metabolic adjustment under extreme stress, although their expression and functional consequences remain poorly constrained (Hu et al., 2025). Some AMD viromes contain AMGs potentially associated with carbon and sulfur metabolism; however, their expression and ecological significance remain largely unresolved (Kieft et al., 2021; Liu et al., 2023). Although SRB-specific phage cultivation and direct virus–host validation remain limited, current evidence suggests that viruses may influence AMD microbial communities, but their direct effects on SRB-mediated sulfate reduction remain speculative. However, their specific effects on SRB-centered sulfate reduction remain largely inferential and require future validation through virus–host linkage, metatranscriptomics, metaproteomics, and in situ functional assays.

Cross-kingdom interactions also contribute to network resilience. Acidophilic fungi tolerate pH 2–3 and secrete extracellular enzymes that degrade complex organic matter into low-molecular-weight compounds, potentially supplying heterotrophic SRB with carbon sources (Ou et al., 2021; Thuy et al., 2025). In contrast, microalgae and higher eukaryotes are limited under extreme acidity; for example, *Acidithiobacillus ferrooxidans* can inhibit acidophilic microalgae through oxidative stress and metabolic disruption, and most microalgae require pre-neutralization of AMD to proliferate, limiting their contribution to sustained carbon input (Wang M. et al., 2025). Consequently, fungi may provide a more persistent contribution to complex carbon degradation in highly acidic systems, whereas sustained microalgal carbon input may be limited to less acidic or pretreated environments.

The effective range of these cellular, community, and habitat-engineering mechanisms defines the resilience boundary of SRB-centered AMD networks. This boundary is unlikely to correspond to a single universal pH or metal threshold. Instead, it emerges from the combined effects of acidification, metal toxicity, carbon limitation, disruption of syntrophic metabolite transfer, oxidative stress, and loss of habitat-engineering capacity. In strongly acidic systems, particularly at pH values below approximately 3, microbial diversity and the abundance of key functional guilds often decline, weakening carbon turnover and redox buffering (Huang et al., 2021). Under such conditions, exogenous carbon addition rarely restores network stability because system limitation shifts from electron donor availability to stress-dominated physiological constraints, including proton and metal toxicity and loss of syntrophic interactions (Zhang et al., 2025a).

The resulting cascade failure may increase the likelihood of a regime shift toward an alternative stable state characterized by persistent acidity, dominance of oxidative guilds, weakened reductive metabolism, and potential hysteresis. Once such a regime shift has occurred, reversing the system may require substantially greater intervention than was required to prevent the transition (Scheffer et al., 2001). Simple substrate amendment is therefore unlikely to be sufficient in severely degraded AMD environments. Recovery may instead require coordinated ecosystem re-engineering, including physicochemical pre-neutralization, reduction of dissolved metal toxicity, restoration of organic-carbon flow, re-establishment of syntrophic and habitat-engineering populations, and stabilization of anoxic microenvironments.

Taken together, these findings indicate that SRB persistence in AMD depends not only on SRB abundance and physiological capacity, but also on the metabolic connectivity, spatial organization, and resilience of the broader microbial community. SRB-mediated sulfate reduction should therefore be understood as an emergent ecosystem function arising from interactions across carbon, sulfur, iron, and nitrogen cycles.

## 4 Regulation of the extracellular microenvironment and maintenance of functional persistence in SRB

In AMD systems, SRB persistence is constrained by insufficient electron donors, acid stress, limited electron transfer, and heavy metal toxicity, and therefore depends not only on cellular tolerance or inoculum density but also on the stability of community-level metabolic interactions and extracellular microenvironments (Chen et al., 2016; Karnachuk et al., 2025). As discussed above, fermentative microorganisms can support SRB by supplying bioavailable carbon substrates, while iron-cycling microorganisms contribute to redox regulation (Demin et al., 2024). However, under persistently low pH, high metal loading, or limited organic matter input, the carbon fluxes, electron fluxes, and spatial organization sustaining these interactions may become destabilized, impairing long-term sulfate reduction (Huang et al., 2021).

In this review, extracellular microenvironments are defined as localized physicochemical niches shaped by EPS, biofilms, mineral surfaces, immobilized carriers, and microbial aggregates. These niches regulate substrate diffusion, pH buffering, electron transfer, metal sequestration, and mineral nucleation. Accordingly, engineering interventions should shift from maximizing short-term sulfate reduction rates toward maintaining the microenvironmental conditions required for persistent SRB function. The following sections discuss five major regulatory strategies: electron donor management, pH buffering, conductive interfaces, mineral–microbe interface regulation, and immobilized reactor design.

### 4.1 Electron donor regulation: carbon release kinetics and electron flux directionality

Electron donors constitute the primary driving force for energy conservation and sulfate reduction in SRB. In AMD systems, the objective of carbon source regulation is not merely to increase total chemical oxygen demand (COD), but rather to favor electron allocation toward sulfate reduction while minimizing electron diversion to competing metabolic pathways and excessive accumulation of fermentation intermediates such as VFAs (Bagheri Novair et al., 2024).

Readily degradable carbon sources accelerate system start-up but often provide limited long-term electron supply stability. In oligotrophic AMD, glycerol amendment rapidly triggered sulfide-mediated removal of Cu^2+^, Zn^2+^, and Cd^2+^, while preferentially enriching incompletely oxidizing SRB such as *Desulfosporosinus* and supporting fermentative microorganisms that generated secondary electron donors (Ilin et al., 2022). However, sulfate reduction rates frequently declined after start-up in systems supplied with rapidly degradable substrates such as industrial syrups, whereas slow-release carbon sources (e.g., alcohol-based emulsified oils) sustained more stable sulfate reduction during long-term operation (Zeng et al., 2019). The metabolic relationship between SRB, fermenters, and MPB shifts along a continuum with the COD/S${\text{O}}_{4}^{2-}$ ratio. At ratios >2, fermenters supply VFAs that sustain SRB activity in a broadly cooperative arrangement. As the ratio approaches stoichiometric limits under carbon limitation, SRB may gain a competitive advantage over MPB for acetate and H_2_ under sulfate-replete conditions due to their thermodynamic advantage, while carbon scarcity simultaneously impairs fermenter hydrolytic efficiency and constrains substrate availability across the community (Chen H. et al., 2023). Although slow-release substrates may not maximize short-term reaction intensity, they stabilize electron availability and reduce metabolic imbalance between fermentation and sulfate reduction, thereby supporting sustained electron flux. In addition, the COD/S${\text{O}}_{4}^{2-}$ ratio strongly influences electron partitioning between sulfate reduction and methanogenesis, altering competition between SRB and methanogens (Cetecioglu et al., 2019; Santos et al., 2022). Thus, readily degradable substrates, slow-release carbon sources, and co-substrate strategies are primarily associated with rapid start-up, long-term stability, and electron-flow coordination, respectively, rather than representing universally superior approaches.

The type of electron donor also influences intracellular electron flux allocation in SRB. For instance, H_2_ generated during lactate oxidation by *Desulfovibrio vulgaris* preferentially drives Fe(III) reduction, whereas pyruvate promotes sulfate reduction through menaquinone-associated electron transfer pathways (Zhou et al., 2017). Therefore, electron donor selection affects not only sulfate reduction rates but also Fe-S mineral formation and the distribution of electrons among alternative terminal electron acceptors.

Natural and low-cost electron donors, including agricultural residues and industrial by-products, provide practical options for long-term AMD treatment but are constrained by the availability of fermentable substrates. In a banana peel fermentation liquor system, 30 d AMD treatment achieved 55.7% S${\text{O}}_{4}^{2-}$ removal and complete Cd^2+^ removal, supported by metabolic coupling between fermentative taxa (*Leptolinea and Sedimentibacter*) and sulfate reducers (*Desulfurispora and Desulfovibrio*) (Gao et al., 2025). A corncob-based SRB–phosphate-reducing bacteria (PRB) system similarly revealed the temporal limitation of lignocellulosic substrates, with sulfate removal progressing through rapid, stable, and declining phases as fermentable components became depleted (Zhang et al., 2025b). Thus, although natural organic substrates can sustain electron supply over extended periods, their long-term performance remains limited by substrate release longevity.

Carbon source engineering in AMD treatment should shift from simple organic supplementation toward electron flux management. Readily degradable substrates facilitate rapid start-up, whereas slow-release carbon sources better sustain long-term electron supply. Co-substrates and carrier-associated carbon sources may further balance start-up efficiency with sustained functionality. However, electron supply is closely coupled to acid–base balance. Short-chain fatty acids generated during fermentation more readily occur in undissociated forms under acidic conditions, allowing membrane diffusion and intracellular acidification (Hao et al., 2014). Consequently, increasing organic loading in low-pH AMD does not necessarily enhance sulfate reduction, as excessive fermentation may promote VFAs accumulation and exacerbate weak-acid stress under acidic conditions (Muyzer and Stams, 2008; Chen et al., 2016). Carbon source engineering should therefore be coordinated with pH buffering and microenvironment stabilization strategies.

### 4.2 pH buffering and construction of the SRB initiation microenvironment

In AMD systems, the inhibitory effects of low pH extend beyond reduced SRB growth to include increased metal bioavailability and disruption of local extracellular conditions. Consequently, engineering pH regulation should focus on establishing buffered extracellular microenvironments that support sustained sulfate reduction rather than simply neutralizing bulk acidity.

External alkalinity input is the most common strategy for creating an SRB initiation window. Alkaline materials such as limestone, dolomite, MgO, and BaCO_3_ consume H^+^ through carbonate dissolution and mineral precipitation, thereby buffering acidity and reducing metal stress (Skousen et al., 2017; Millán-Becerro et al., 2023). Iron-based materials provide an additional advantage by simultaneously regulating pH, redox potential, and electron supply. Under acidic conditions, zero-valent iron corrosion consumes H^+^ and releases Fe^2+^ and H_2_, which can support hydrogenotrophic SRB, lower Eh, and promote Fe–S mineral formation. Representative studies using iron–carbon microelectrolysis, Fe^0^/Fe^2+^-enhanced immobilized SRB, and sulfidated zero-valent iron all indicate that iron-based materials can expand the initiation window for SRB under acidic, metal-rich conditions (Li et al., 2023; Chen et al., 2024; Shao et al., 2026). However, both alkaline and iron-based amendments may lose effectiveness over time because of alkalinity depletion, surface passivation, or precipitate accumulation.

Biological and spatial strategies further expand the effective pH boundary for SRB establishment. Indigenous or selectively enriched acid-tolerant SRB can maintain sulfate reduction under moderately acidic conditions, whereas gradual acidification during pre-cultivation may activate community-level stress responses and improve SRB acclimation (Alazard et al., 2010; Sánchez-Andrea et al., 2013; Zhang et al., 2025a). Alkalinity-generating microorganisms, such as ureolytic bacteria, can also reshape local chemical fluxes by producing ammonia and carbonate, thereby creating buffered zones that facilitate SRB colonization (Peng S.-X. et al., 2025). Biofilms and microbial aggregates further stabilize buffered extracellular microniches by restricting proton diffusion and maintaining favorable local pH conditions for SRB colonization, even when the surrounding bulk environment remains acidic (Lens et al., 1993; Ito et al., 2002; Diez-Ercilla et al., 2019).

Once SRB become established, sulfate reduction itself generates alkalinity, reinforcing local pH recovery and creating a positive feedback that further stabilizes the extracellular microenvironment (Neculita et al., 2007). Hydrochemical modeling and pilot-scale studies further suggest that biologically generated alkalinity can become a decisive factor governing long-term stability in acidic waters with limited background buffering capacity (Zhou et al., 2022; Ma et al., 2024). More complex biological interfaces, such as microalgae–SRB stratified systems, may further reinforce extracellular buffering by coupling photosynthetic H^+^ consumption with sulfate-reduction-driven alkalinity generation (Chen L. Y. et al., 2025). These processes create a positive feedback loop in which initial buffering enables sulfate reduction, while sulfate reduction progressively stabilizes the local extracellular microenvironment.

Overall, pH regulation in AMD-SRB systems should be viewed as a staged process involving external buffering, biological acclimation, spatial microenvironment protection, and endogenous alkalinity generation. Alkaline and iron-based materials provide the initial conditions for SRB establishment, biofilms and immobilized structures protect local niches, and established sulfate reduction supports long-term pH stabilization. Thus, successful SRB colonization depends on maintaining buffered extracellular microenvironments rather than simply increasing bulk pH.

### 4.3 Conductive interfaces and reinforcement of electron transfer networks

Sustained sulfate reduction depends not only on electron-donor availability but also on efficient electron distribution within microbial communities. In AMD systems, low pH, high metal loads, and mineral precipitates can increase electron-transfer resistance among SRB, minerals, and syntrophic partners, thereby reducing overall electron utilization. Because syntrophic communities rely on multiple forms of metabolite-mediated and extracellular electron exchange, conductive interfaces provide an engineering means of reinforcing these pre-existing interaction networks. Rather than introducing fundamentally new metabolic pathways, conductive materials can improve the efficiency, spatial range, and stability of electron transfer within SRB-centered communities.

Exogenous conductive materials, including activated carbon, biochar, magnetite, and Fe_3_O_4_, reduce electron transfer resistance and reshape microbial electron networks. Powdered activated carbon (PAC) enhances expression of e-pili and cytochrome genes, reinforcing electron-sharing among *Desulfovibrio, Methanothrix*, and *Geobacter*, whereas Fe_3_O_4_ primarily provides an external conductive pathway (Shu et al., 2025). Biogenic FeS minerals, such as mackinawite and FeS nanoparticles, form natural conductive matrices supporting long-range EET and interspecies electron exchange (Kondo et al., 2015; Murugan and Okamoto, 2025).

Conductive materials can also modify interactions between SRB and other functional microbes, particularly by influencing electron partitioning between sulfate reduction and methanogenesis. In upflow anaerobic sludge blanket (UASB) reactors, granular activated carbon (GAC) enhanced both sulfate reduction and methane production while promoting syntrophic interactions between Desulfovibrio and Methanosaeta (Chen L. et al., 2023). Rather than establishing new metabolic pathways, conductive materials strengthen existing syntrophic electron-transfer networks, thereby improving electron utilization and cooperative metabolism within the community.

Although iron-based conductive materials (Fe^0^ and derivatives) provide auxiliary reducing capacity, modulate redox potential, and drive up to 98.2% sulfate removal via Fe^2+^ release and Fe–S formation (Li et al., 2023, 2024), their long-term application is limited by surface passivation from metal sulfide and oxide deposition. Overcoming this bottleneck requires strategies to control biofilm thickness and prevent precipitate crystallization. Alternatively, constructing integrated electrode–material–SRB networks allows SRB to directly acquire electrons from electrodes, effectively supplementing conventional carbon-derived electron sources (Saini et al., 2026).

However, enhanced electron transfer may redirect electrons toward methanogenesis, potentially reducing sulfide production and metal immobilization. For example, biochar altered community electron partitioning, diverting approximately 26.6% of electrons from sulfate reduction toward methane production and consequently decreasing metal sulfide formation (Tsui et al., 2022). Therefore, the effectiveness of conductive materials in AMD should be assessed based on electron flux partitioning, sulfate reduction contribution, and final metal fixation, rather than on the abundance of electron-transfer genes or methane yield alone.

Overall, conductive materials should be regarded as engineering tools for regulating community-scale electron fluxes rather than simply enhancing conductivity. Their practical value lies in directing electrons toward sulfate reduction while minimizing undesirable diversion to competing pathways such as methanogenesis. Future reactor design should therefore emphasize controllable electron allocation instead of maximizing conductivity alone.

### 4.4 Mineral–microbe interfaces: balancing metal immobilization and cellular activity maintenance

In AMD systems, SRB must simultaneously sustain sulfate reduction and immobilize metals under conditions enriched in Fe^3+^, Al^3+^, and other toxic metal species. Mineral–microbe interfaces are therefore not merely passive attachment sites but dynamic microenvironments where biofilm formation, extracellular electron transfer, mineral transformation, and metal sulfide nucleation interact to regulate SRB activity and metal immobilization. Minerals may provide protection, nutrients, and electron-transfer pathways, but they may also release toxic elements or induce oxidative stress, highlighting their context-dependent roles in AMD remediation (Dong et al., 2022).

The key challenge is that metal sulfide formation does not necessarily translate into sustained SRB activity. More important than the total amount of precipitate is where precipitation occurs. When FeS, ZnS, CdS, and related precipitates nucleate directly on SRB cell surfaces, they may block substrate diffusion, restrict metabolite exchange, interfere with electron transfer, and ultimately cause cell inactivation. Thus, effective interface engineering should not simply aim to maximize metal sulfide precipitation, but should spatially redirect mineral formation away from sensitive cell envelopes toward extracellular matrices or abiotic carrier surfaces.

This spatial pattern is largely governed by mineral surface properties. The surface thermodynamic and electrochemical properties of minerals constitute the important determinants of SRB colonization and subsequent interfacial reactions. According to extended DLVO theory, microbial adhesion is governed by the combined effects of interfacial free energy, electrostatic interactions, and electron donor–acceptor interactions among surface functional groups (Du et al., 2022). Iron oxides generally promote biofilm formation, whereas aluminum oxide coatings generated under acidic conditions may reduce microbial attachment. Low-pH environments also alter EPS conformation and surface charge, introducing non-DLVO interactions that influence preferential colonization sites and local accumulation of reduced metabolites and metal ions, thereby shaping the spatial distribution of metal sulfide precipitation.

**Figure 3:**
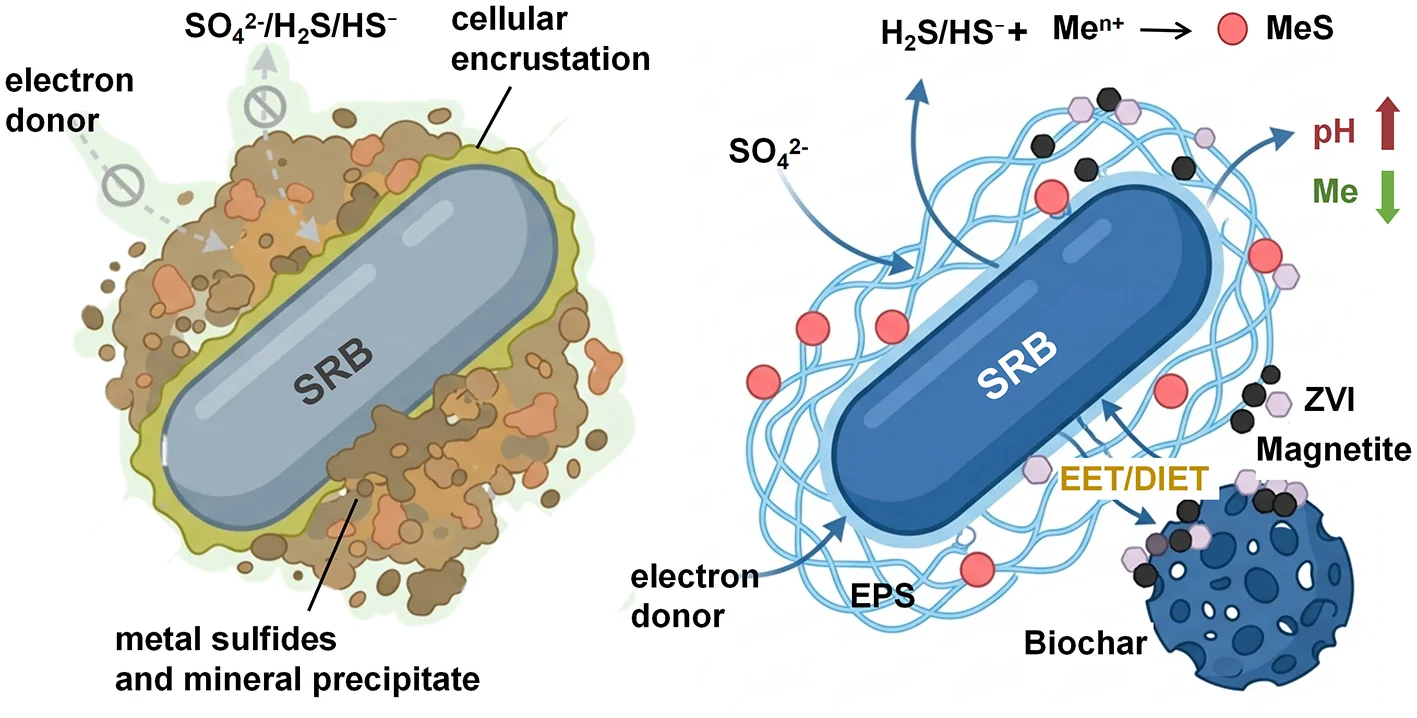
Spatial decoupling of metal sulfide precipitation from SRB cell surfaces. Direct precipitation of metal sulfides on SRB envelopes can cause cell encrustation, mass-transfer blockage, and metabolic inactivation. EPS matrices and functional carriers provide alternative nucleation sites that redirect metal sulfide formation away from cell surfaces. This ex-situ precipitation strategy helps maintain substrate diffusion, electron transfer, and SRB viability during AMD remediation.

EPS provides the first biological interface for this spatial decoupling. Functional groups such as carboxyl, phosphate, hydroxyl, and amino moieties can complex metal ions and promote off-membrane nucleation within the extracellular matrix, reducing local metal toxicity and limiting direct cell encrustation (Gan et al., 2023; Chen J. et al., 2025). Functional carriers, including biochar, magnetite, activated carbon, and other mineral-based materials, further expand abiotic nucleation sites and act as sinks for metal sulfide precipitation. As illustrated in Figure 3, the combined action of EPS and engineered carriers promotes ex-situ precipitation, thereby separating metal immobilization from cellular inactivation and preserving mass-transfer pathways.

Redox-active minerals further regulate SRB activity by mediating electron transfer and mineral transformation. The Fe(III)/Fe(II) cycle can couple iron reduction with sulfate reduction, while minerals containing iron, sulfur, or manganese may function as electron donors, acceptors, or conductive media that sustain microscale electron fluxes (Dong et al., 2022). Biogenic FeS generated by SRB is particularly important because it serves both as a metal immobilization product and as a potential electron-conducting phase that can enhance extracellular electron transfer (Kondo et al., 2015; Ke D. D. et al., 2023). However, excessive FeS accumulation may clog pores, passivate carrier surfaces, and impair mass transfer. Therefore, the beneficial role of FeS depends on maintaining dynamic mineral regeneration rather than allowing uncontrolled precipitate accumulation.

Overall, mineral–microbe interface engineering in AMD-SRB systems should shift from cell-surface mineralization toward controlled ex-situ interfacial mineralization. Mineral surface modification, EPS-guided nucleation, carrier-mediated precipitation, and FeS-mediated electron transfer can decouple metal immobilization from SRB inhibition. However, key trade-offs remain: EPS-associated precipitation may compete with carrier-based precipitation, enhanced electron transfer may divert electrons away from sulfate reduction, and long-term mineral accumulation may cause passivation or pore blockage. Future studies should combine microscale in situ characterization, electrochemical monitoring, and long-term reactor assessment to clarify how interface evolution controls SRB persistence and AMD remediation stability.

### 4.5 Immobilized carriers and reactor systems

While electron donor regulation, local buffering, electron-transfer network reinforcement, and mineral–microbe interface optimization primarily target microscale environments surrounding SRB, these functions are difficult to sustain at scale without engineering support. Immobilized carriers and reactor systems provide not only physical substrates for microbial growth but also spatial compartmentalization, slow substrate release, and hydraulic control, thereby enabling the persistence of microscale microenvironmental functions at the engineering scale.

Immobilized carriers create protective microenvironmental layers through encapsulation, granulation, or biofilm formation, allowing SRB to maintain metabolic activity under acidic and metal-stressed conditions. For example, hydrogel-embedded SRB maintained sulfate reduction rates of 0.71–0.86 g S${\text{O}}_{4}^{2-}\cdot$L^−1^·d^−1^ when temperature dropped from 24 °C to 15 °C, whereas non-immobilized consortia nearly lost activity (Yin et al., 2025). Similarly, cold-tolerant SRB consortia retained sulfate reduction rates of 1000–4500 mg·L^−1^ at 11.7 °C following immobilization (Virpiranta et al., 2022). These results indicate that immobilization not only preserves biomass but also buffers cells against direct perturbations from temperature, pH, and metal pulses.

Modern immobilized carriers are increasingly multifunctional, providing microbial attachment, carbon supply, buffering capacity, and electron transfer facilitation. For instance, maifanite carriers enhance S${\text{O}}_{4}^{2-}$ removal and stabilize system pH (Dong et al., 2020); nanoscale zero-valent iron (nZVI) in carriers improves electron availability and metal reduction (Wang et al., 2021); immobilized SRB granules on *Rhodopseudomonas spheroides*–activated lignite combine structural support, slow carbon release, and biomass retention (Di et al., 2022); hydrothermal carbon carriers maintain high sulfate reduction efficiency over multiple cycles (Feng et al., 2026). Such multifunctional designs integrate microscale protective functions, enabling synergistic effects of electron supply, substrate slow release, and localized buffering.

However, immobilization has inherent limitations. Denser particles increase diffusion resistance for substrates (S${\text{O}}_{4}^{2-}$, VFAs, H_2_S), and long-term mineral deposition or pore blockage can reduce mass transfer, affecting SRB activity (Sakr et al., 2023; Zhang et al., 2025b). Therefore, carrier design must balance protection and substrate accessibility to ensure microenvironmental functions are maintained without diffusion constraints.

At the unit scale, permeable reactive barriers (PRBs), anaerobic wetlands, and other passive systems are widely applied. Long-term stability depends on the dynamic interplay among slow carbon release, alkalinity maintenance, and mineral deposition. For example, a corncob PRB treating AMD at pH 2.5 achieved 73.4% S${\text{O}}_{4}^{2-}$ removal; however, carbon depletion and pore blockage gradually reduced system performance (Zhang et al., 2025b). Periodic carbon supplementation or slow-release substrates can delay decline but cannot fully prevent long-term deterioration (Luo et al., 2025). Microenvironmental regulation relies on spatial stratification: organic substrates supply electrons, alkaline materials maintain buffering, SRB mediate sulfate reduction and metal immobilization, and mineral precipitates concentrate in defined zones, achieving functional division and dynamic balance.

At the system scale, continuous-flow and multi-stage coupled reactors are emerging as preferred solutions for high-load AMD treatment and metal recovery. Spatially decoupling Fe oxidation, SRB reduction, and sulfur-oxidizing bacteria (SOB) processes allows each unit to operate under optimal conditions, minimizing interference. Pilot-scale systems have demonstrated that 12m^3^/d coupled Fe-oxidation, SRB reduction, and SOB oxidation reactors can achieve >99% Cu^2+^ removal while significantly reducing operational costs (Hu et al., 2022). In counter-current fluidized bed systems, multi-metal recovery rates are maintained above 90% (Kumar and Pakshirajan, 2021, 2022). UASB systems exhibit high sulfate-reducing activity under both acidic and neutral conditions (Rojas-Torreblanca et al., 2025). These results indicate that continuous-flow and multi-stage reactors can systematically sustain microenvironmental functions through hydraulic retention time control and granular sludge maintenance.

In summary, immobilized carriers and reactor systems represent complementary strategies for maintaining SRB microenvironments across scales. Carriers provide local protection and function integration; passive systems sustain carbon and buffering supply; and continuous-flow and multi-stage reactors enable spatial decoupling and long-term controllable operation. Their synergistic application illustrates a shift in SRB engineering design from “instantaneous reaction enhancement” toward “microenvironmental stability maintenance,” offering a feasible pathway for long-term AMD remediation.

### 4.6 Summary: from process intensification to system stability maintenance

SRB-based AMD remediation systems often experience performance decline because electron supply, acid–base balance, mineral precipitation, electron transfer, and mass transport become progressively decoupled during long-term operation (Muyzer and Stams, 2008; Hao et al., 2014). Increasing a single process, such as carbon addition, conductivity, or mineral precipitation, may improve short-term performance but can also trigger secondary constraints, including accumulation of VFAs, electron diversion, cell encrustation, surface passivation, or pore blockage (Tsui et al., 2022; Ke et al., 2024).

Therefore, engineering design should prioritize system stability rather than isolated process intensification. Electron donor regulation sustains electron flux; pH buffering expands the SRB initiation window; conductive materials improve electron transfer but require control of electron partitioning; mineral–microbe interface regulation separates metal immobilization from cellular inhibition; and immobilized systems preserve these functions across operational scales. Because these engineering strategies differ substantially in engineering maturity, scalability, operational requirements, and long-term performance, a systematic comparison is necessary to support strategy selection for practical AMD remediation.

**Figure 4:**
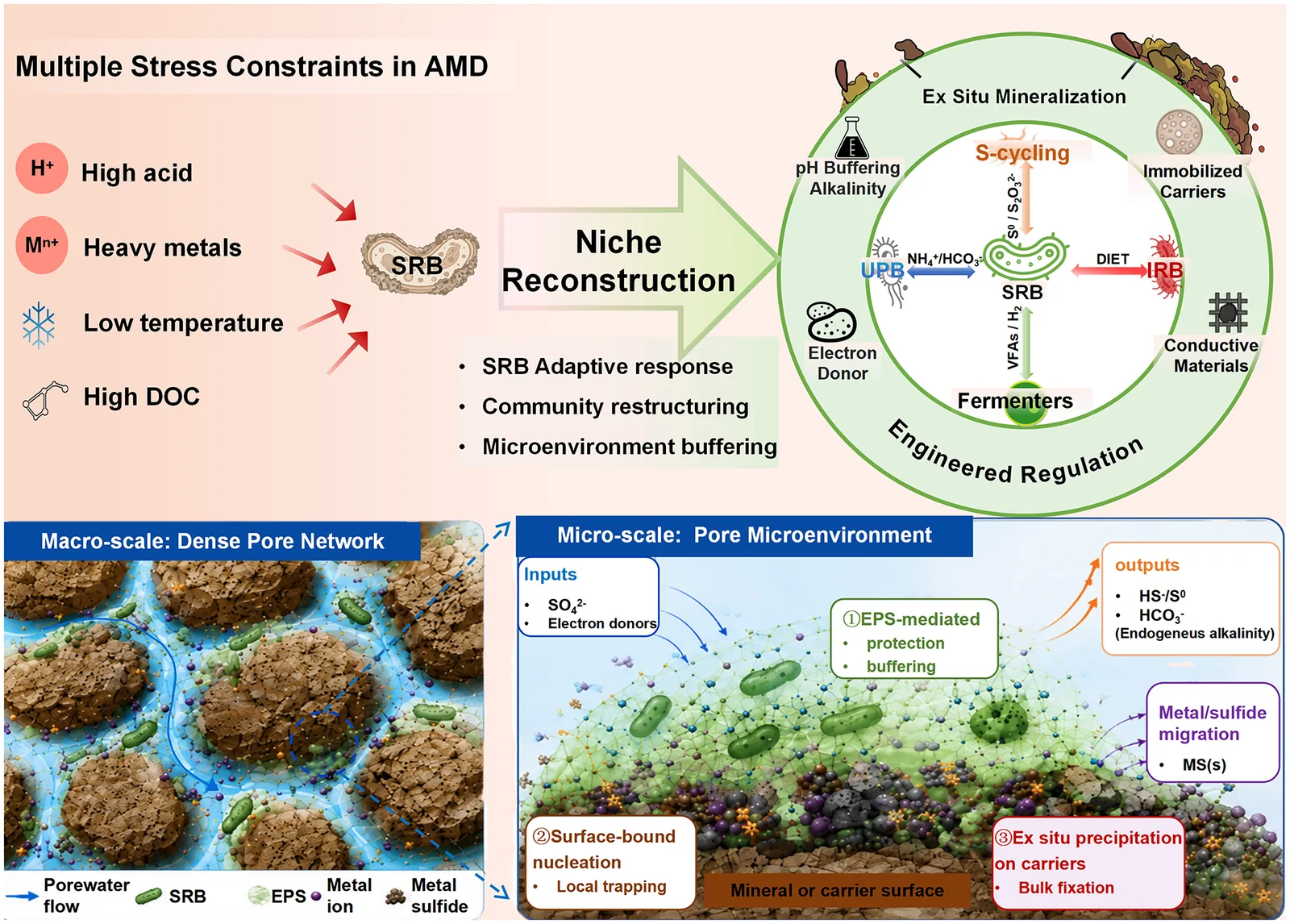
Niche reconstruction and engineered regulation for sustaining SRB activity in AMD systems. AMD stressors disrupt sulfate-reducing microenvironments by altering pH, redox conditions, and substrate availability. SRB persistence is maintained through niche reconstruction and reinforced by engineered regulation, which stabilizes electron transfer processes, microbial interactions, and mineral transformation pathways, enabling sustained sulfate reduction under extreme conditions.

Together, these strategies reconstruct extracellular microenvironments that maintain the physicochemical conditions required for persistent sulfate reduction. Figure 4 summarizes the proposed mechanisms by which extracellular microenvironment stabilization supports long-term SRB activity. For practical comparison, Table 3 further compares representative stabilization strategies in terms of sulfate removal performance, engineering maturity, scalability, operational cost, long-term stability, and major limitations.

**Table 3:** Engineering strategies for maintaining extracellular microenvironment stability in SRB-mediated AMD remediation.

Strategy	Sulfate removal performance	Engineering maturity	Scalability	Operational cost	Long-term stability	Major limitation	References
Rapid-release carbon sources	High during start-up (up to ~94%)	Bench–pilot	Medium (continuous carbon supply required)	Medium	Transient	VFAs accumulation; carbon depletion; continuous carbon dosing required	Ilin et al., 2022; Mafane et al., 2025
Slow-release carbon substrates	Moderate–High (typically 20%−78%; up to ~69% in passive systems)	Pilot–field	High (widely applicable to passive systems)	Low	Stable	Carbon exhaustion; heterogeneous release; replacement difficulty	Zeng et al., 2019; Wang et al., 2024
Fe-based materials	High enhancement (up to ~94%)	Pilot	Medium–High (readily integrated into composite reactors)	Medium	Conditionally stable	Passivation; mineral precipitation	An et al., 2023; Zhang et al., 2024
Conductive materials	Moderate enhancement	Laboratory	Low (limited field-scale validation)	Medium	Unverified	Limited field validation; uncertain engineering benefits	Thisani et al., 2021; Zhang et al., 2024
Immobilization carriers	High (up to ~94%; >200 d continuous operation)	Continuous-flow/Pilot	Medium (engineering feasible but preparation-intensive)	Medium	Stable	Diffusion limitation; carrier preparation complexity	An et al., 2023; Yin et al., 2025
Passive reactive systems (PRBs, wetlands)	Moderate–High (typically 44%−69%)	Field-scale	High (widely demonstrated at field scale)	Low	Stable	Hydraulic clogging; HRT loss; media aging	Anungstri et al., 2023; Wang et al., 2024
Multi-stage coupled reactors	Very high (up to ~99%)	Pilot–field	Medium (engineering feasible but operationally complex)	Medium–High	Stable	Operational complexity; process integration	Thisani et al., 2022

Despite these advances, most studies remain limited to short-term, laboratory-scale, or simulated AMD systems, whereas long-term operational data under authentic AMD conditions remain scarce. In particular, the coupled relationships among carbon-release longevity, localized pH gradients, FeS dynamic evolution, pore blockage, and microbial succession have yet to be quantitatively integrated into a unified model (Diez-Ercilla et al., 2019; Luo et al., 2025). Future research should combine long-term continuous-flow experiments, reactive transport modeling, microscale interfacial characterization, and microbial community dynamics analysis to develop an integrated understanding of how electron flux, acid–base balance, mineral phase evolution, and mass transport processes interact during long-term operation (Craig et al., 2021; Zhou et al., 2022). Such a systems-level perspective may help shift SRB engineering from empirical optimization toward more predictive and sustainable reactor design.

## 5 Conclusions and perspectives

### 5.1 Conclusions

Sulfate-reducing bacteria (SRB) have long been recognized as key drivers of acid mine drainage (AMD) bioremediation because they simultaneously promote sulfate removal, metal sulfide precipitation, and alkalinity generation. This review highlights that sustained sulfate reduction in AMD depends on interactions among SRB physiology, environmental stress, microbial community processes, and extracellular conditions rather than on SRB tolerance alone.

Multiple AMD stressors, including acidity, heavy metal toxicity, low temperature, and electron-donor limitation, act synergistically by simultaneously increasing cellular maintenance energy requirements and reducing energy acquisition. Rather than independently inhibiting sulfate reduction, these stressors progressively contract the effective metabolic niche available for sustained SRB activity, providing a mechanistic explanation for the discrepancy between laboratory performance and field-scale remediation.

As the metabolic niche contracts, SRB become increasingly reliant on microbial interactions that stabilize electron supply, carbon turnover, redox buffering, and coupled iron–sulfur–nitrogen cycling. Consequently, sustained sulfate reduction should be regarded as an emergent ecosystem function arising from resilient microbial interaction networks rather than as the activity of individual SRB populations alone.

Engineering strategies therefore function primarily by reconstructing extracellular microenvironments that expand the effective metabolic niche and stabilize microbial interaction networks. These approaches target distinct but interconnected constraints, including electron availability, proton stress, interfacial reactions, and spatial organization, thereby improving electron transfer, mass transport, and mineral transformation to support long-term functional persistence.

Collectively, this framework suggests that successful AMD bioremediation requires a shift from maximizing short-term sulfate reduction toward rebuilding the metabolic niches and ecological networks that sustain SRB function. Because multiple stressors progressively constrain SRB energy availability and physiological flexibility, future strategies should integrate multi-omics, spatially resolved analyses, long-term field observations, and predictive modeling to identify resilience boundaries and design more stable, adaptive SRB-based remediation systems.

### 5.2 Moving forward

Although considerable progress has been made in understanding SRB-mediated AMD remediation, several critical knowledge gaps remain. Most current studies are based on short-term laboratory experiments or simplified AMD simulations, whereas long-term observations under authentic field conditions are still scarce. Consequently, the coupled relationships among carbon-source depletion, pH dynamics, mineral precipitation, pore clogging, and microbial succession remain poorly understood.

A major challenge for future research is to link microbial processes with the physicochemical evolution of the extracellular microenvironment. Advanced approaches such as single-cell analysis, spatial transcriptomics, NanoSIMS, in situ electrochemical measurements, and integrated multi-omics techniques provide opportunities to resolve microbial spatial organization, metabolic interactions, and electron-transfer pathways at previously inaccessible scales.

From an engineering perspective, future efforts should move beyond maximizing sulfate reduction rates and instead focus on maintaining long-term system stability. Developing resilient microbial consortia, adaptive carrier materials, and predictive models that integrate electron fluxes, acid–base balance, mineral phase evolution, and mass-transfer processes will be essential for achieving reliable and scalable AMD bioremediation.
