# Supplementary material for: Sulfate-reducing bacteria in acid mine drainage: ecological constraints, microbial networks, and functional persistence
Source: Front Microbiol. 2026 Aug 11;17:1910720. doi: 10.3389/fmicb.2026.1910720 (PMC13505485; doi:10.3389/fmicb.2026.1910720)
Supplement: Supplementary file 1 [file Table_1.docx]

Supplementary Table S1. Major microbial functional guilds interacting with sulfate-reducing bacteria (SRB) in AMD ecosystems and their ecological effects on sulfate reduction.

| **Functional guild** | **Main ecological role** | **Interaction with SRB** | **Overall effect** | **Representative taxa** | **References** |
| --- | --- | --- | --- | --- | --- |
| **Fermentative bacteria** | Hydrolysis and fermentation of complex organics to H_2_, lactate, formate, ethanol, acetate,and volatile fatty acids (VFAs) | Metabolic syntrophy; supply H₂, formate, lactate, acetate and VFAs to SRB | **Positive** | Clostridium, Sedimentibacter, Anaerovorax | (Liang et al., 2023; Wang et al., 2023) |
| Syntrophic fatty-acid oxidizers | Oxidation of propionate/butyrate to acetate ^+^ H_2_ under low H_2_ partial pressure | Obligate metabolic syntrophy; SRB maintain low H₂/formate levels that enable fatty-acid oxidation | Positive | *Syntrophus, Syntrophomonas, Pelotomaculum* | (Kieft et al., 2021) |
| **Iron-reducing bacteria (FeRB)** | Dissimilatory Fe(III) reduction to Fe(II) coupled to organic matter oxidation | Cooperative but context-dependent; release mineral-bound sulfate and promote Fe–S precipitation, with possible metal mobilization or immobilization depending on mineral pathways | Generally positive; geochemically context-dependent | Shewanella,Geobacter | (Ahmed et al., 2021; Sun et al., 2025) |
| **Sulfur-disproportionating bacteria (SDB)** | Disproportionation of elemental sulfur or thiosulfate to H_2_S and SO_4_^2-^ | Indirect facilitation via sulfur disproportionation; sustain sulfidic microenvironments and metal-sulfide precipitation under carbon limitation | Indirectly positive; context-dependent | Desulfurivibrio | (Zou et al., 2023) |
| **Methanogens** | Methanogenesis from CO_2_/H_2_ or acetate | Competitive for H_2_ and acetate; usually suppressed under acidic, sulfate-rich AMD but may increase after neutralization/sulfate depletion | **Primarily negative; conditionally bidirectional** | Methanosaeta, Methanoculleus, Methanosarcina | (Méndez et al., 2023; J. et al., 2024) |
| **Viruses (phages)** | Host lysis and potential AMG-mediated metabolic modulation | Community-level regulation through host lysis and AMGs; SRB-specific host range remains uncertain | **Context-dependent** | AMD viral operational taxonomic units (vOTUs) | (Kieft et al., 2021; Liu et al., 2023) |
| **Sulfur-oxidizing bacteria (SOB)** | Oxidation of H_2_S/S^0^ to SO_4_^2^- | Antagonistic; re-oxidize sulfide/sulfur intermediates and regenerate acidity | Bulk-scale negative; potentially complementary at redox interfaces | Acidithiobacillus,Thiobacillus | (Whaley-Martin et al., 2023) |
| **Iron-oxidizing bacteria (FeOB)** | Oxidation of Fe(II) to Fe(III) with proton production | Antagonistic; oxidize Fe(II) to Fe(III),with acidity promoted by Fe(III) hydrolysis / mineralization | Bulk-scale negative; coexistence possible in structured biofilms | Leptospirillum,Acidithiobacillus ferrooxidans | (Ren et al., 2025) |
| **Nitrate-reducing bacteria (NRB)** | Reduction of NO_3_^-^ to NH_4_^+^or N_2_ | Competitive for shared electron donors; nitrate/nitrite metabolism may suppress sulfide accumulation or shift SRB activity | Neutral to negative; context-dependent | Paracoccus denitrificans, Pseudomonas stutzeri, Thiobacillus denitrificans | (Marques and Rodrigues, 2025) |
